# Supplementary material for: Transplacental SARS-CoV-2 protein ORF8 binds to complement C1q to trigger fetal inflammation
Source: EMBO J. 2024 Oct 10;43(22):10. doi: 10.1038/s44318-024-00260-9 (PMC11574245; doi:10.1038/s44318-024-00260-9)
Supplement: Supplementary file 1 — Table EV1 [file 44318_2024_260_MOESM1_ESM.docx]

**Table EV1. Summary of SARS-CoV-2 detection in maternal-fetal samples from COVID-19 pregnant cohort**

| COVID-19  pregnant patients | Trimester | Maternal | | Infant | |
| --- | --- | --- | --- | --- | --- |
|  |  | **ORF8** | **ddPCR^a^** | **ORF8** | **ddPCR^a^** |
| COV02 | **3** | No | No | No | Yes |
| COV04 | **3** | Yes | No | N/A | No |
| COV06 | **3** | No | No | Yes | Yes |
| COV08 | **3** | Yes | No | Yes | Yes |
| COV10 | **2** | No | Yes | N/A | No |
| COV11 | **3** | Yes | No | Yes | No |
| COV12 | **3** | Yes | No | No | No |
| COV14 | **3** | Yes | No | Yes | No |
| COV15 | **2** | Yes | Yes | Yes | No |
| COV16 | **3** | Yes | Yes | Yes | No |
| COV17 | **1** | No | Yes | Yes | Yes |
| COV18 | **3** | No | No | Yes | No |
| COV20 | **1** | Yes | No | No | No |
| COV22 | **2** | Yes | Yes | Yes | No |
| COV26 | **3** | Yes | No | Yes | No |
| COV27 | **3** | Yes | No | No | No |
| COV29 | **3** | No | No | N/A | Yes |
| COV31 | **3** | No | No | Yes | No |
| COV32 | **3** | Yes | No | Yes | No |
| COV33 | **3** | Yes | No | No | No |
| COV34 | **3** | No | No | Yes | No |
| COV35 | **2** | Yes | No | No | Yes |
| COV36 | **2** | Yes | Yes | Yes | No |
| % (n) | | **65.2% (15/23)** | **26% (6/23)** | **70% (14/20)** | **26% (6/23)** |

^a^ddPCR results includes N1 and N1+N2 positivity. N/A indicates specimen not available for testing.
