# Supplementary material for: Transplacental SARS-CoV-2 protein ORF8 binds to complement C1q to trigger fetal inflammation
Source: EMBO J. 2024 Oct 10;43(22):10. doi: 10.1038/s44318-024-00260-9 (PMC11574245; doi:10.1038/s44318-024-00260-9)
Supplement: Supplementary file 2 — Table EV2 [file 44318_2024_260_MOESM2_ESM.docx]

**Table EV2. Average of SARS-CoV-2 infection clearance to delivery in positive samples from COVID-19 pregnant cohort**

| COVID-19 pregnancy | | Maternal | | Infant | |
| --- | --- | --- | --- | --- | --- |
| Average infection to delivery in (days) | | | | | |
| Trimester | **Per trimester (N)** | **ORF8 + (N)** | **ddPCR+ (N)** | **ORF8+ (N)** | **ddPCR+ (N)** |
| First | 202 (2) | 216 (1) | 188 (1) | 188 (1) | 188 (1) |
| Second | 105 (5) | 104.5 (4) | 106.8 (4) | 106.7 (3) | 98 (1) |
| Third | 34.3 (16) | 44.9 (12) | 59 (1) | 29.5 (10) | 23.8 (4) |
