# Supplementary material for: Transplacental SARS-CoV-2 protein ORF8 binds to complement C1q to trigger fetal inflammation
Source: EMBO J. 2024 Oct 10;43(22):10. doi: 10.1038/s44318-024-00260-9 (PMC11574245; doi:10.1038/s44318-024-00260-9)
Supplement: Supplementary file 3 — Table EV3 [file 44318_2024_260_MOESM3_ESM.docx]

**Table EV3. Predicted interacting residues and binding interactions between C1qA and SARS-CoV-2 ORF8 peptides.**

| **Interacting amino acid (AA) residues** | | | **Distance, Å** | **No. of specific binding interactions** | | |
| --- | --- | --- | --- | --- | --- | --- |
| **C1qA - Globular** | **SARS-CoV-2 ORF8** | |  | **H-bond** | **Salt Bridge** | **van der Waals** |
|  | **AA residue** | **Peptide ID** |  |  |  |  |
| **Met104** | **Gln18** | **Peptide #4** | 1.0 | 0 | 0 | 4 |
|  | **Glu19** | **Peptide #4** | 1.4 | 0 | 0 | 1 |
|  | **His112** | **Peptide #25*** | 0.20 | 0 | 0 | 36 |
|  | **Val114** | **Peptide #25*** | 1.7 | 0 | 0 | 1 |
| **Gly105** | **Gln18** | **Peptide #4** | 1.2 | 0 | 0 | 9 |
|  | **Val114** | **Peptide #25*** | 1.2 | 0 | 0 | 13 |
| **Trp147** | **His40** | **Peptide #10** | 1.0 | 0 | 0 | 13 |
|  | **Phe104** | **Peptide #25** | 1.3 | 0 | 0 | 1 |
| **Glu148** | **Ile39** | **Peptide #10** | 2.0 | 0 | 0 | 1 |
|  | **His40** | **Peptide #10** | 1.6 | 0 | 0 | 3 |
| **Arg158** | **Phe41** | **Peptide #10** | 1.5 | 0 | 0 | 1 |
|  | **Phe104** | **Peptide #25** | 1.4 | 0 | 0 | 1 |
|  | Tyr105 | **Peptide #25** | 0.50 | 0 | 0 | 23 |
|  | **Tyr111** | **Peptide #25** | 2.0 | 0 | 0 | 1 |
| **Gly159** | **Phe41** | **Peptide #10** | 1.5 | 0 | 0 | 2 |
|  | **His112** | **Peptide #25*** | 0.80 | 0 | 0 | 12 |
| **Lys197** | **Gln18** | **Peptide #4** | 1.7 | 0 | 0 | 1 |
|  | Pro36 | **Peptide #10** | 2.3 | 1 | 0 | 0 |
| **His203** | **Ile39** | **Peptide #10** | 1.3 | 0 | 0 | 3 |
|  | **Phe41** | **Peptide #10** | 1.6 | 0 | 0 | 2 |
|  | **Phe104** | **Peptide #25** | 1.4 | 0 | 0 | 2 |

*AA in bold face: residue that has more than one (1) predicted contact or interaction with the other protein*

**AA residue is proximal to the SARS-CoV-2 ORF8 peptide by one (1) or three (3) residue/s*
