# Supplementary material for: Transplacental SARS-CoV-2 protein ORF8 binds to complement C1q to trigger fetal inflammation
Source: EMBO J. 2024 Oct 10;43(22):10. doi: 10.1038/s44318-024-00260-9 (PMC11574245; doi:10.1038/s44318-024-00260-9)
Supplement: Supplementary file 4 — Table EV4 [file 44318_2024_260_MOESM4_ESM.docx]

**Table EV4. SARS-CoV-2 ORF8 AA residues with predicted interactions at multiple sites in C1qA.**

| **Interacting amino acid (AA) residues** | | | **Distance, Å** | **No. of specific binding interactions** | | |
| --- | --- | --- | --- | --- | --- | --- |
| **SARS-CoV-2 ORF8** | | **C1qA-Globular** |  | **H-bond** | **Salt Bridge** | **van der Waals** |
| **Peptide ID** | **AA residue** |  |  |  |  |  |
| **Peptide #4** | **Gln18** | **Met104** | 1.0 | 0 | 0 | 4 |
| **Peptide #4** |  | **Gly105** | 1.2 | 0 | 0 | 9 |
| **Peptide #4** |  | Val108 | 2.1 | 1 | 0 | 0 |
| **Peptide #4** |  | Ile110 | 1.7 | 1 | 0 | 1 |
| **Peptide #4** |  | **Lys197** | 1.7 | 0 | 0 | 1 |
| **Peptide #4** | **Glu19** | Pro103 | 0.50 | 0 | 0 | 8 |
| **Peptide #4** |  | **Met104** | 1.4 | 0 | 0 | 1 |
| **Peptide #4** |  | Arg128 | 0.90 | 1 | 1 | 4 |
| **Peptide #10** | Pro36 | **Lys197** | 2.3 | 1 | 0 | 0 |
| **Peptide #10** | **Pro38** | Gln192 | 1.4 | 0 | 0 | 10 |
| **Peptide #10** |  | Gly202 | 1.2 | 0 | 0 | 3 |
| **Peptide #10** | **Ile39** | Val130 | 1.6 | 0 | 0 | 4 |
| **Peptide #10** |  | **Glu148** | 2.0 | 0 | 0 | 1 |
| **Peptide #10** |  | **His203** | 1.3 | 0 | 0 | 3 |
| **Peptide #10** | **His40** | **Trp147** | 1.0 | 0 | 0 | 13 |
| **Peptide #10** |  | **Glu148** | 1.6 | 0 | 0 | 3 |
| **Peptide #10** |  | Asn172 | 2.2 | 0 | 0 | 1 |
| **Peptide #10** | **Phe41** | **Arg158** | 1.5 | 0 | 0 | 1 |
| **Peptide #10** |  | **Gly159** | 1.5 | 0 | 0 | 2 |
| **Peptide #10** |  | Gly190 | 1.6 | 0 | 0 | 3 |
| **Peptide #10** |  | **His203** | 1.6 | 0 | 0 | 2 |
| **Peptide #25** | **Phe104** | **Trp147** | 1.3 | 0 | 0 | 1 |
| **Peptide #25** |  | **Arg158** | 1.4 | 0 | 0 | 1 |
| **Peptide #25** |  | **His203** | 1.4 | 0 | 0 | 2 |
| **Peptide #25** | Tyr105 | **Arg158** | 0.50 | 0 | 0 | 23 |
| **Peptide #25** | **Tyr111** | **Arg158** | 2.0 | 0 | 0 | 1 |
| **Peptide #25** |  | Gln160 | 1.1 | 0 | 0 | 10 |
| **Peptide #25*** | **His112** | **Met104** | 0.20 | 0 | 0 | 36 |
| **Peptide #25*** |  | **Gly159** | 0.80 | 0 | 0 | 12 |
| **Peptide #25*** | **Val114** | **Met104** | 1.7 | 0 | 0 | 1 |
| **Peptide #25*** |  | **Gly105** | 1.2 | 0 | 0 | 13 |

*AA in bold face: residue that has more than one (1) predicted contact or interaction with the other protein*

**AA residue is proximal to the SARS-CoV-2 ORF8 peptide by one (1) or three (3) residue/s*
