# Supplementary material for: Transplacental SARS-CoV-2 protein ORF8 binds to complement C1q to trigger fetal inflammation
Source: EMBO J. 2024 Oct 10;43(22):10. doi: 10.1038/s44318-024-00260-9 (PMC11574245; doi:10.1038/s44318-024-00260-9)
Supplement: Supplementary file 12 — Source data Fig. 7 [file 44318_2024_260_MOESM12_ESM.zip › Figure_7_B_and_C.pptx]

## Slide 1
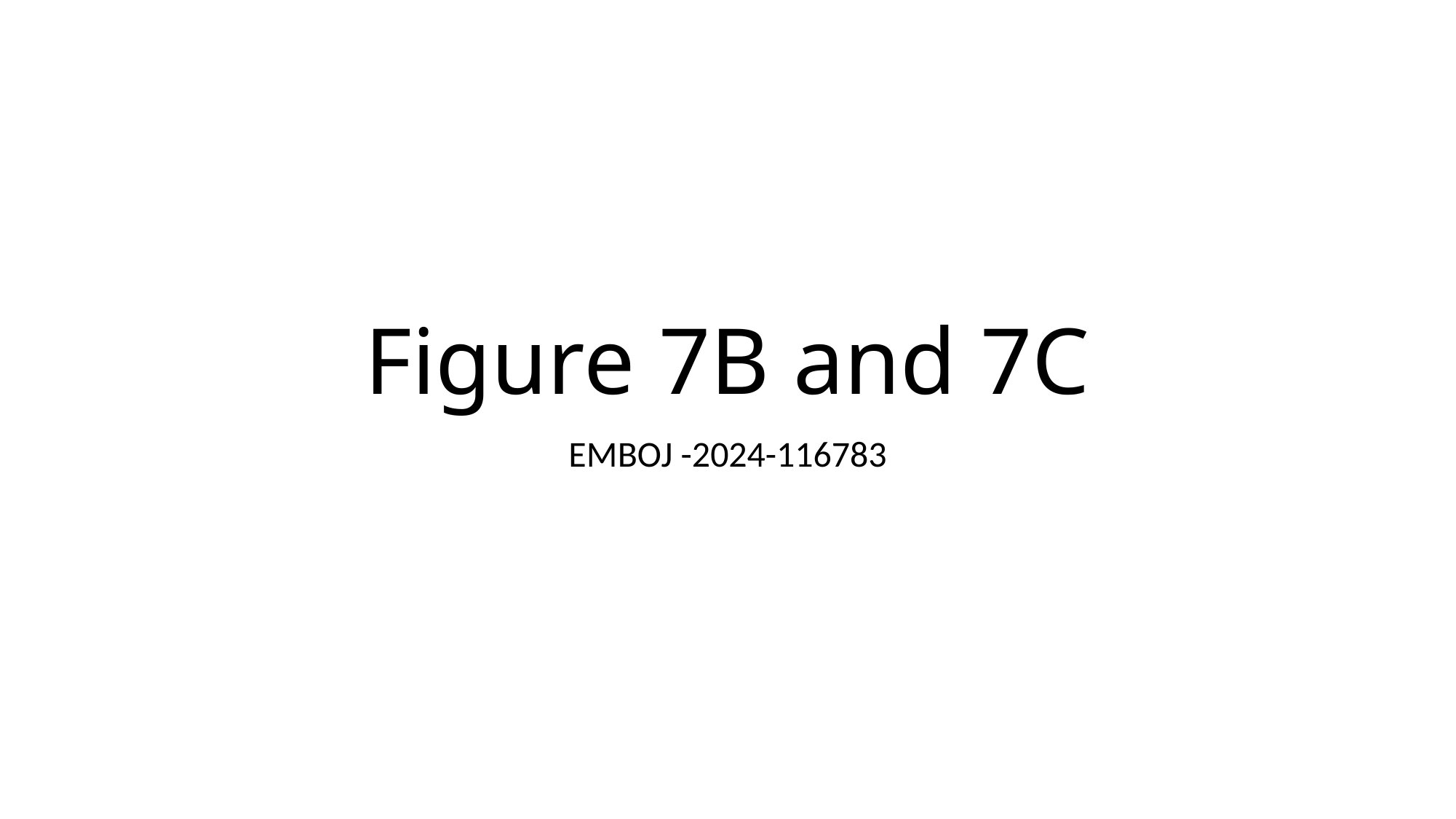

# Figure 7B and 7C
EMBOJ -2024-116783

## Slide 2
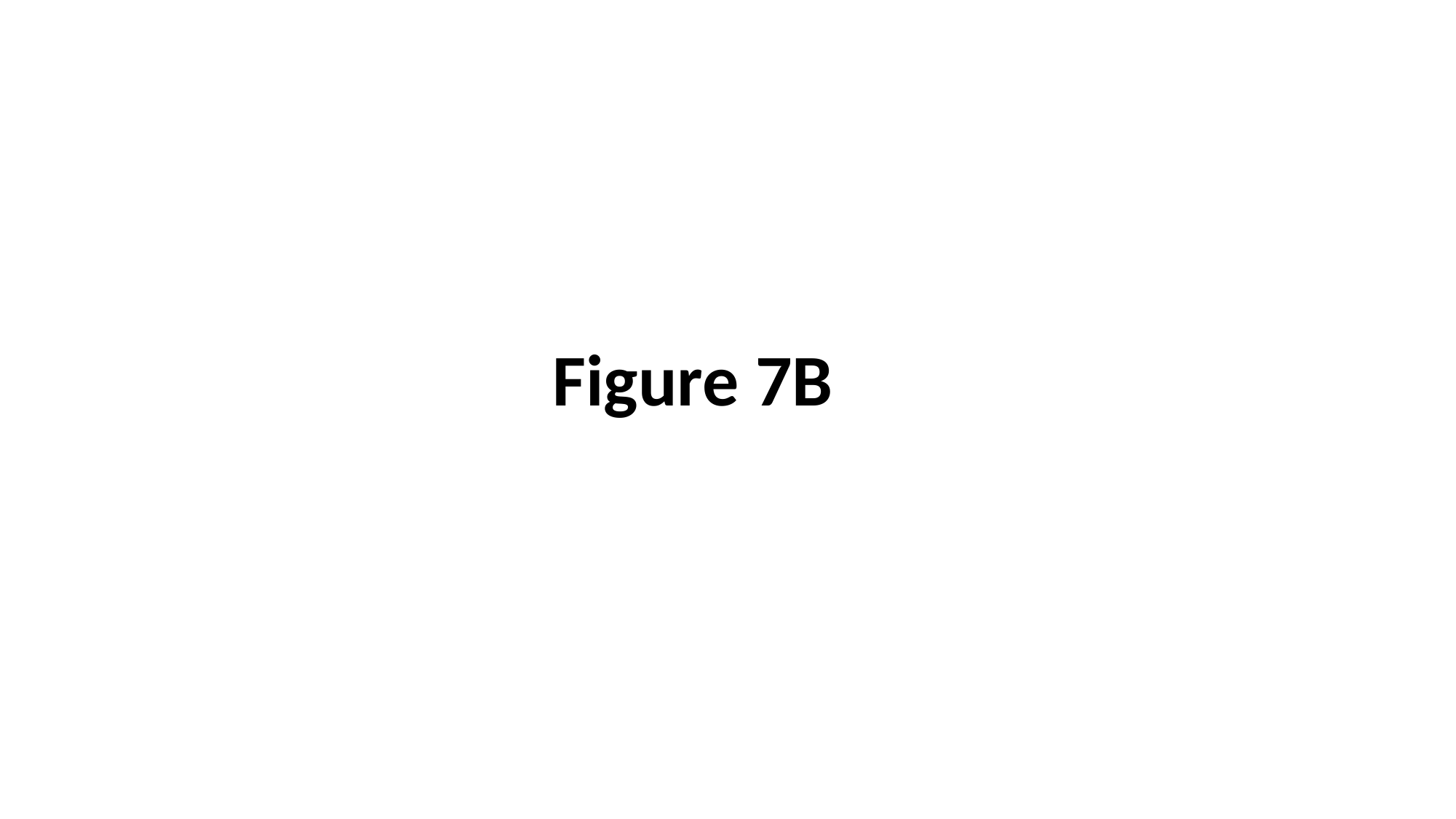

Figure 7B

## Slide 3
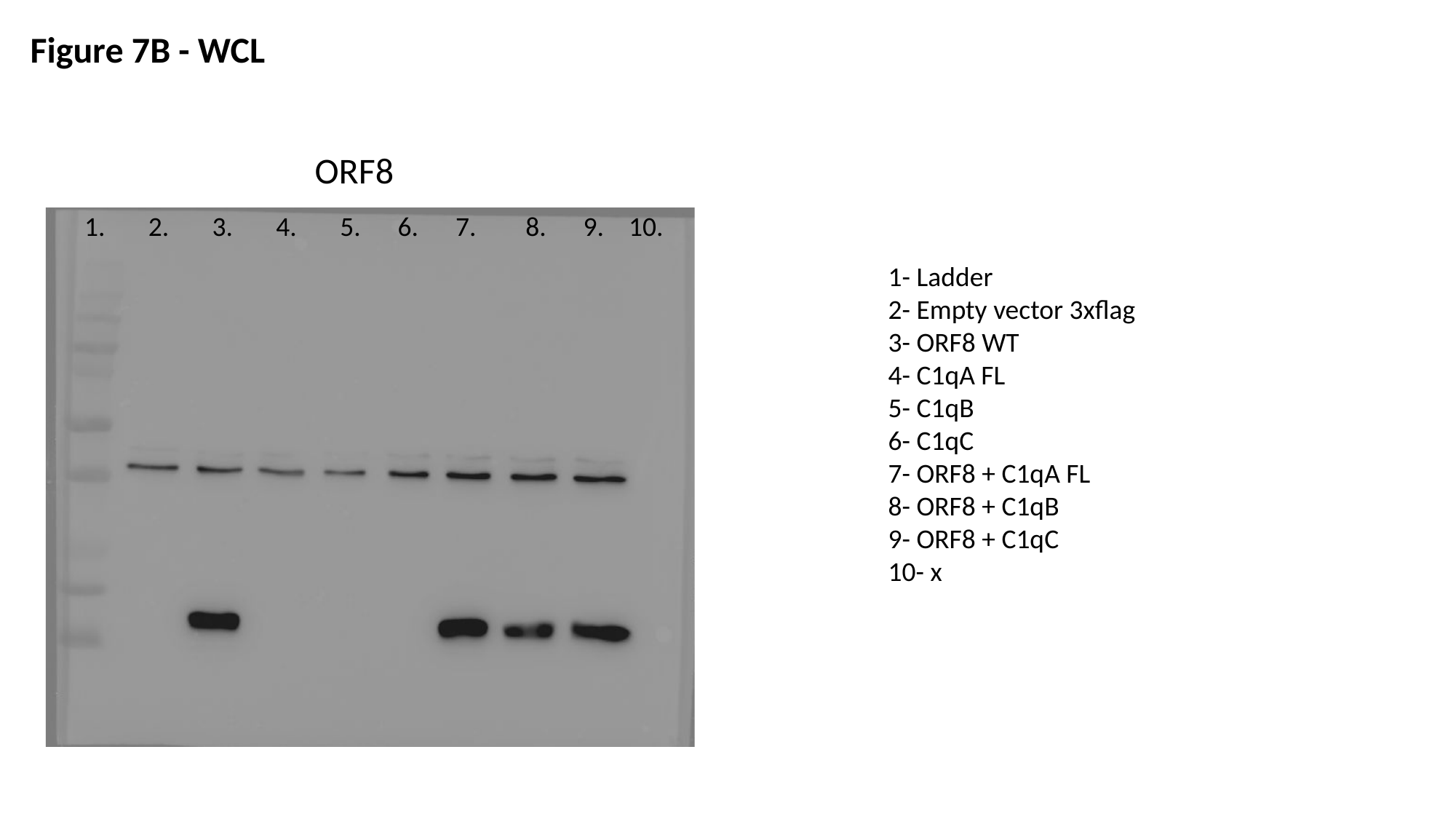

Figure 7B - WCL
ORF8
1. 2. 3. 4. 5. 6. 7. 8. 9. 10.
1- Ladder
2- Empty vector 3xflag
3- ORF8 WT
4- C1qA FL
5- C1qB
6- C1qC
7- ORF8 + C1qA FL
8- ORF8 + C1qB
9- ORF8 + C1qC
10- x

## Slide 4
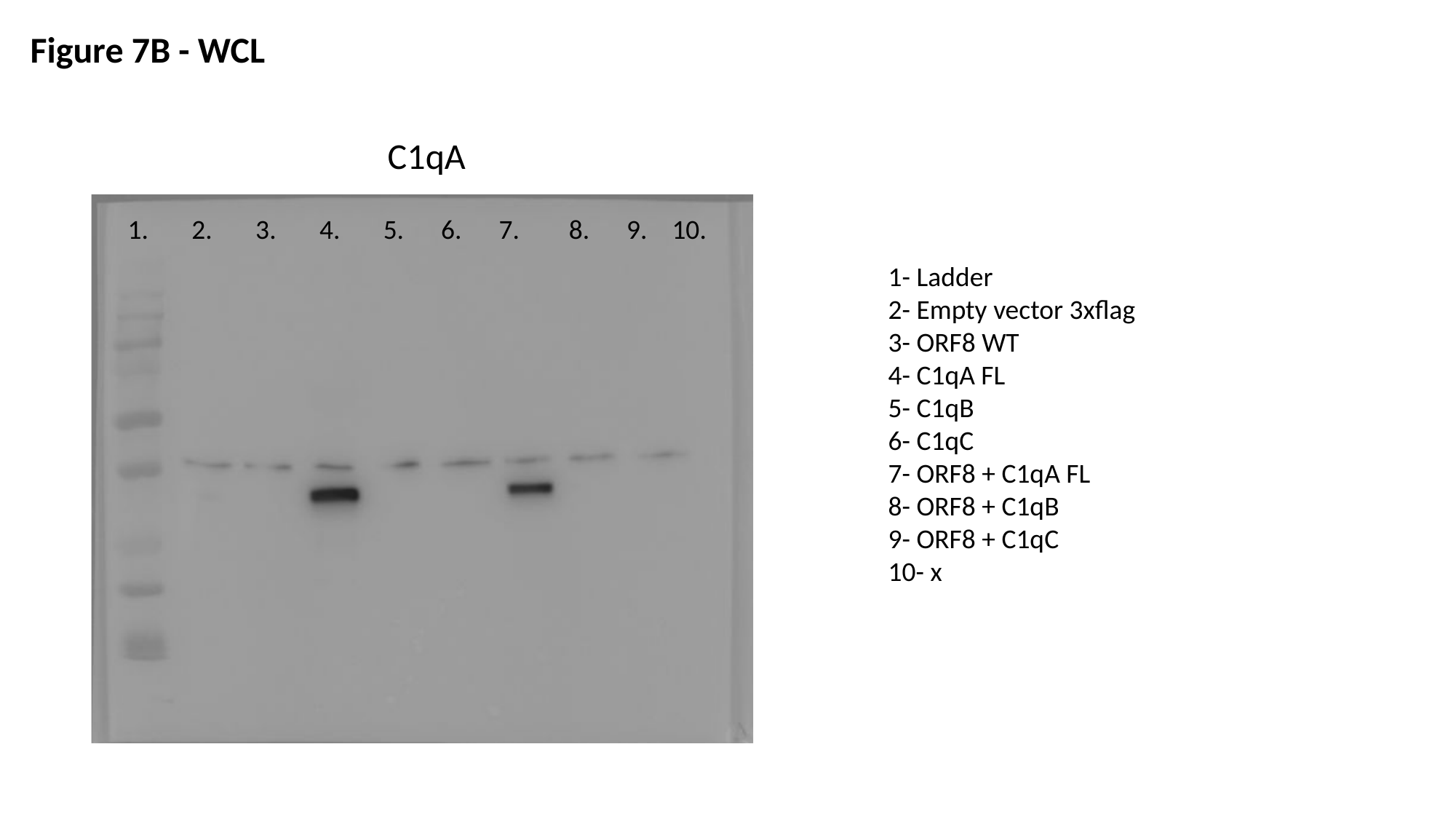

Figure 7B - WCL
C1qA
1. 2. 3. 4. 5. 6. 7. 8. 9. 10.
1- Ladder
2- Empty vector 3xflag
3- ORF8 WT
4- C1qA FL
5- C1qB
6- C1qC
7- ORF8 + C1qA FL
8- ORF8 + C1qB
9- ORF8 + C1qC
10- x

## Slide 5
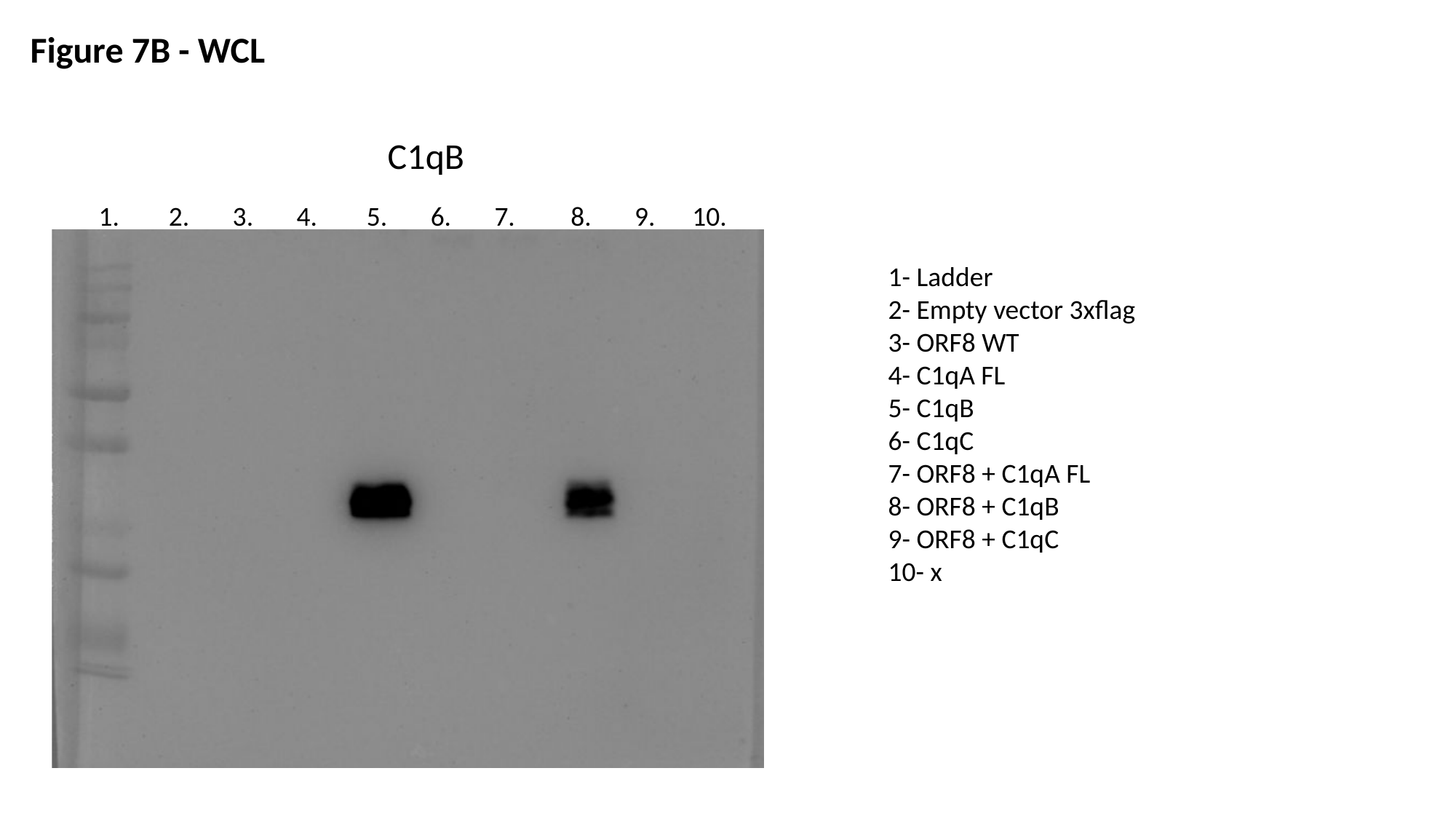

Figure 7B - WCL
C1qB
1. 2. 3. 4. 5. 6. 7. 8. 9. 10.
1- Ladder
2- Empty vector 3xflag
3- ORF8 WT
4- C1qA FL
5- C1qB
6- C1qC
7- ORF8 + C1qA FL
8- ORF8 + C1qB
9- ORF8 + C1qC
10- x

## Slide 6
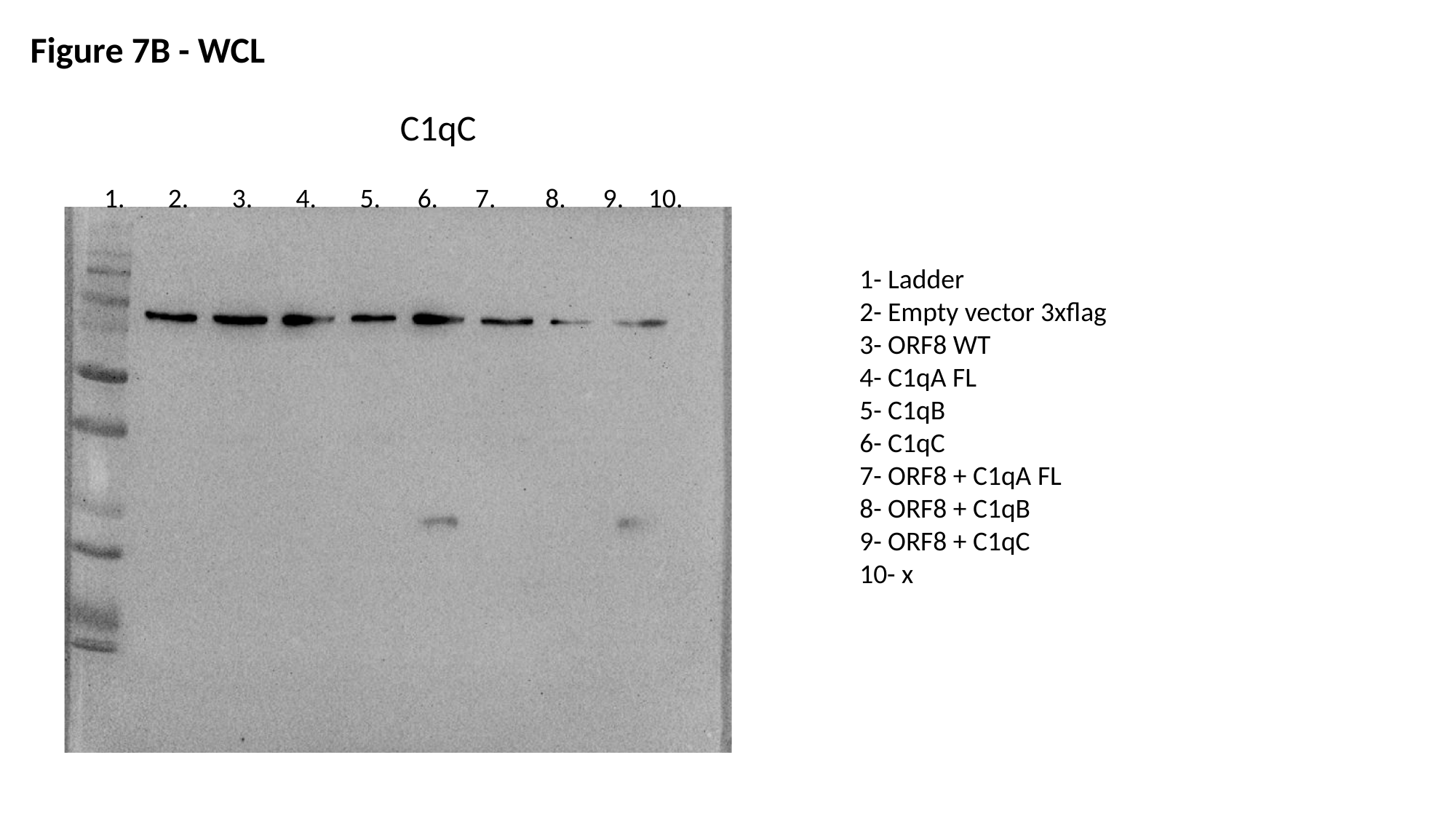

Figure 7B - WCL
C1qC
1. 2. 3. 4. 5. 6. 7. 8. 9. 10.
1- Ladder
2- Empty vector 3xflag
3- ORF8 WT
4- C1qA FL
5- C1qB
6- C1qC
7- ORF8 + C1qA FL
8- ORF8 + C1qB
9- ORF8 + C1qC
10- x

## Slide 7
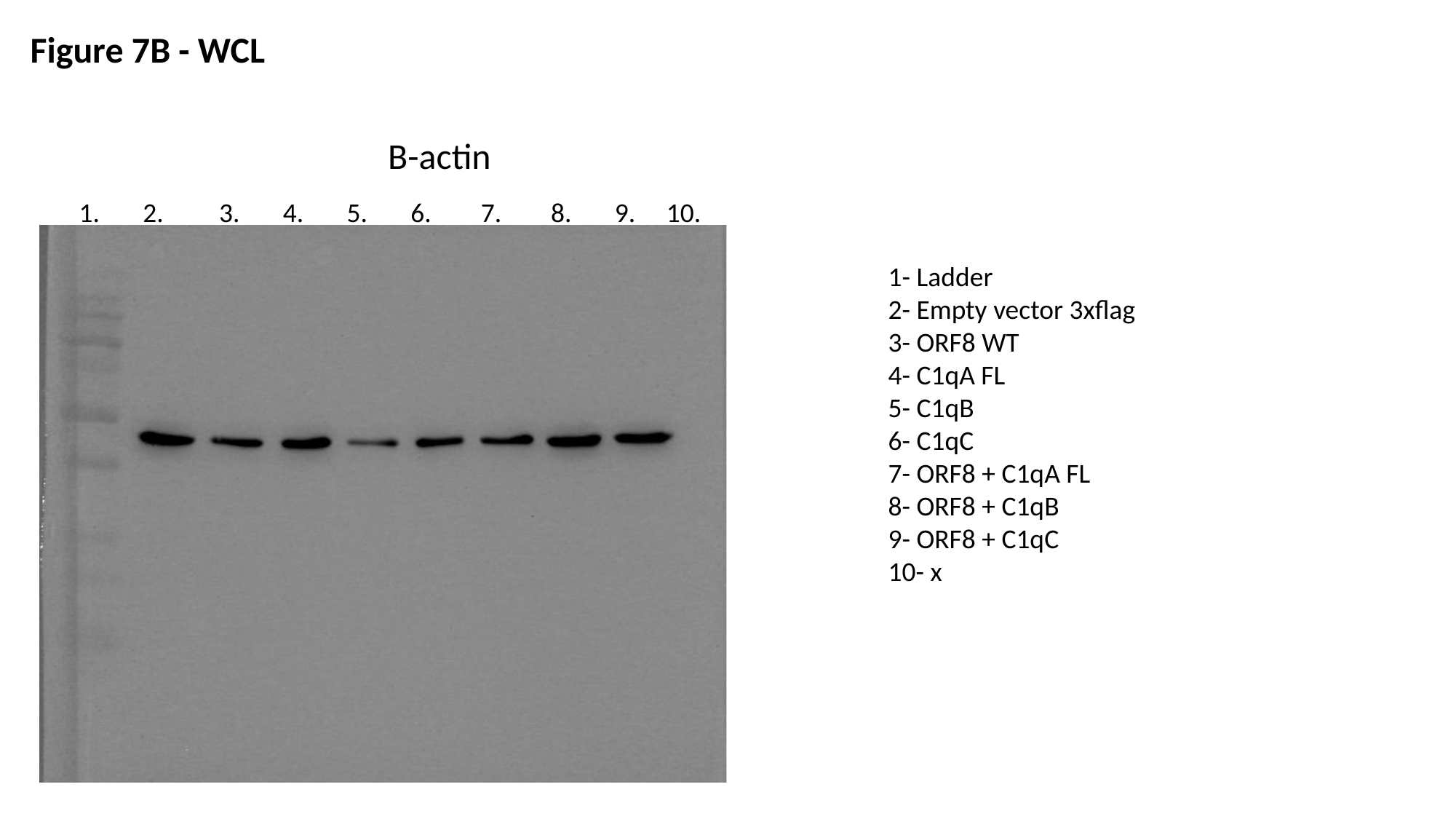

Figure 7B - WCL
B-actin
1. 2. 3. 4. 5. 6. 7. 8. 9. 10.
1- Ladder
2- Empty vector 3xflag
3- ORF8 WT
4- C1qA FL
5- C1qB
6- C1qC
7- ORF8 + C1qA FL
8- ORF8 + C1qB
9- ORF8 + C1qC
10- x

## Slide 8
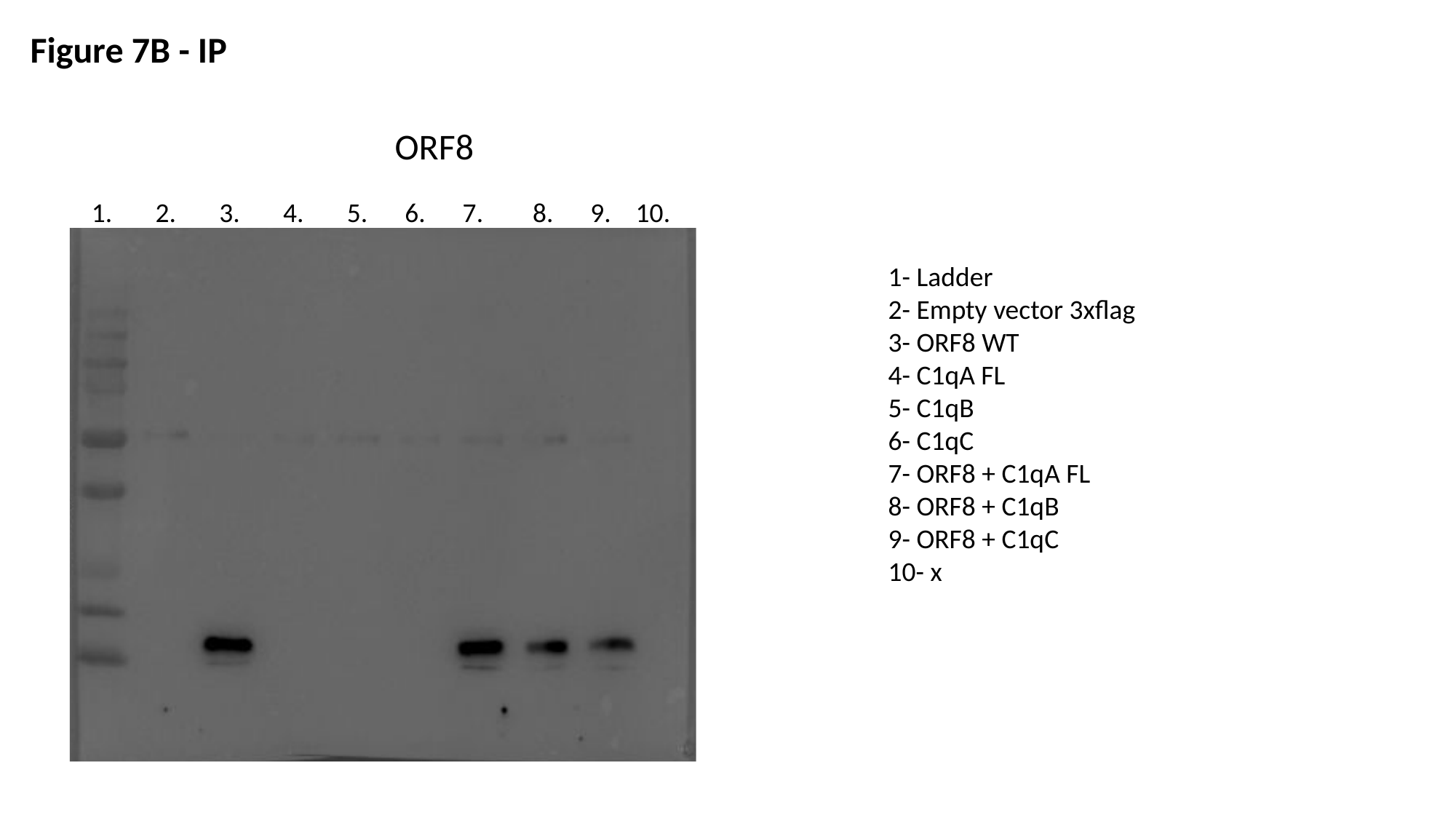

Figure 7B - IP
ORF8
1. 2. 3. 4. 5. 6. 7. 8. 9. 10.
1- Ladder
2- Empty vector 3xflag
3- ORF8 WT
4- C1qA FL
5- C1qB
6- C1qC
7- ORF8 + C1qA FL
8- ORF8 + C1qB
9- ORF8 + C1qC
10- x

## Slide 9
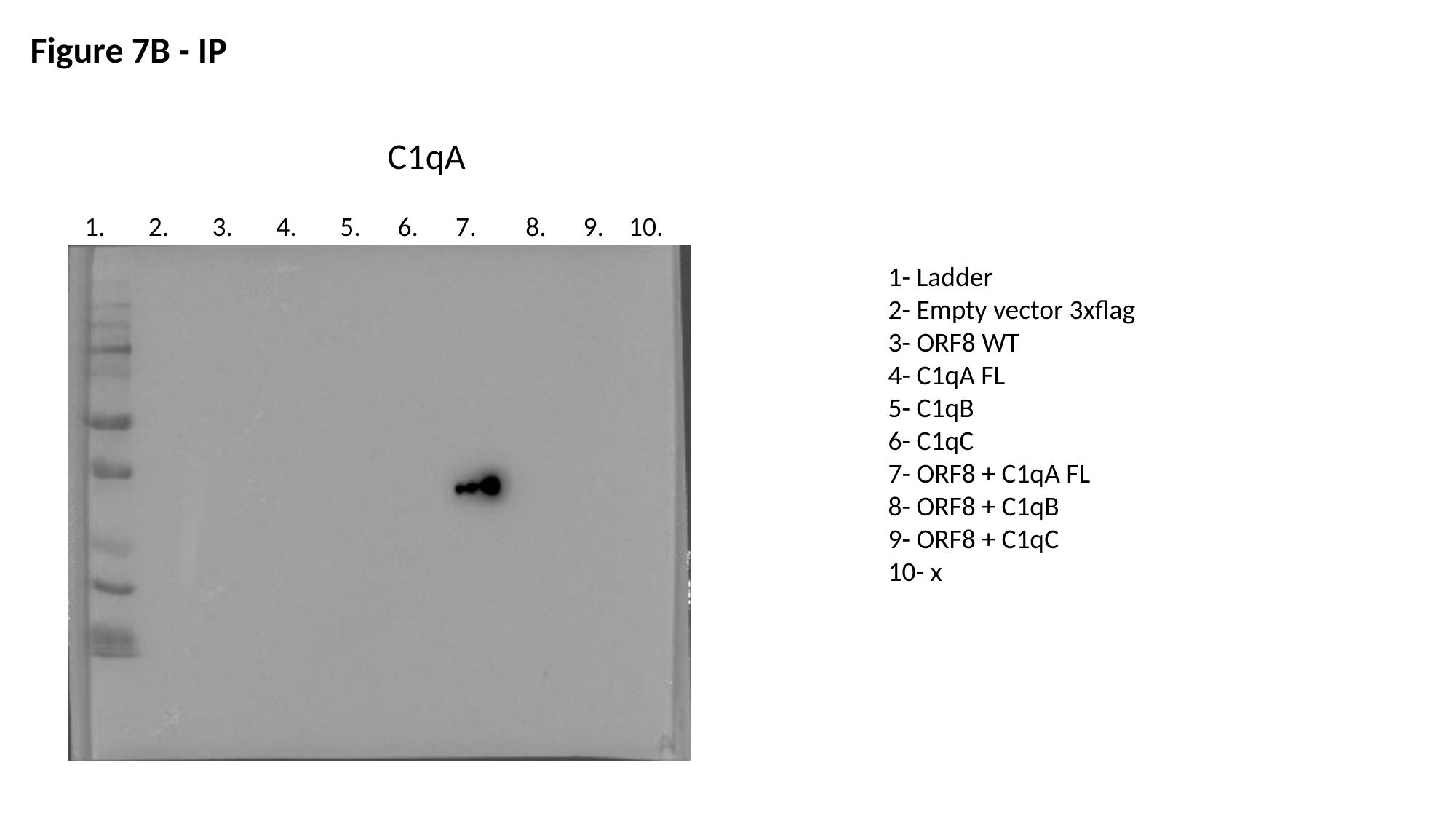

Figure 7B - IP
C1qA
1. 2. 3. 4. 5. 6. 7. 8. 9. 10.
1- Ladder
2- Empty vector 3xflag
3- ORF8 WT
4- C1qA FL
5- C1qB
6- C1qC
7- ORF8 + C1qA FL
8- ORF8 + C1qB
9- ORF8 + C1qC
10- x

## Slide 10
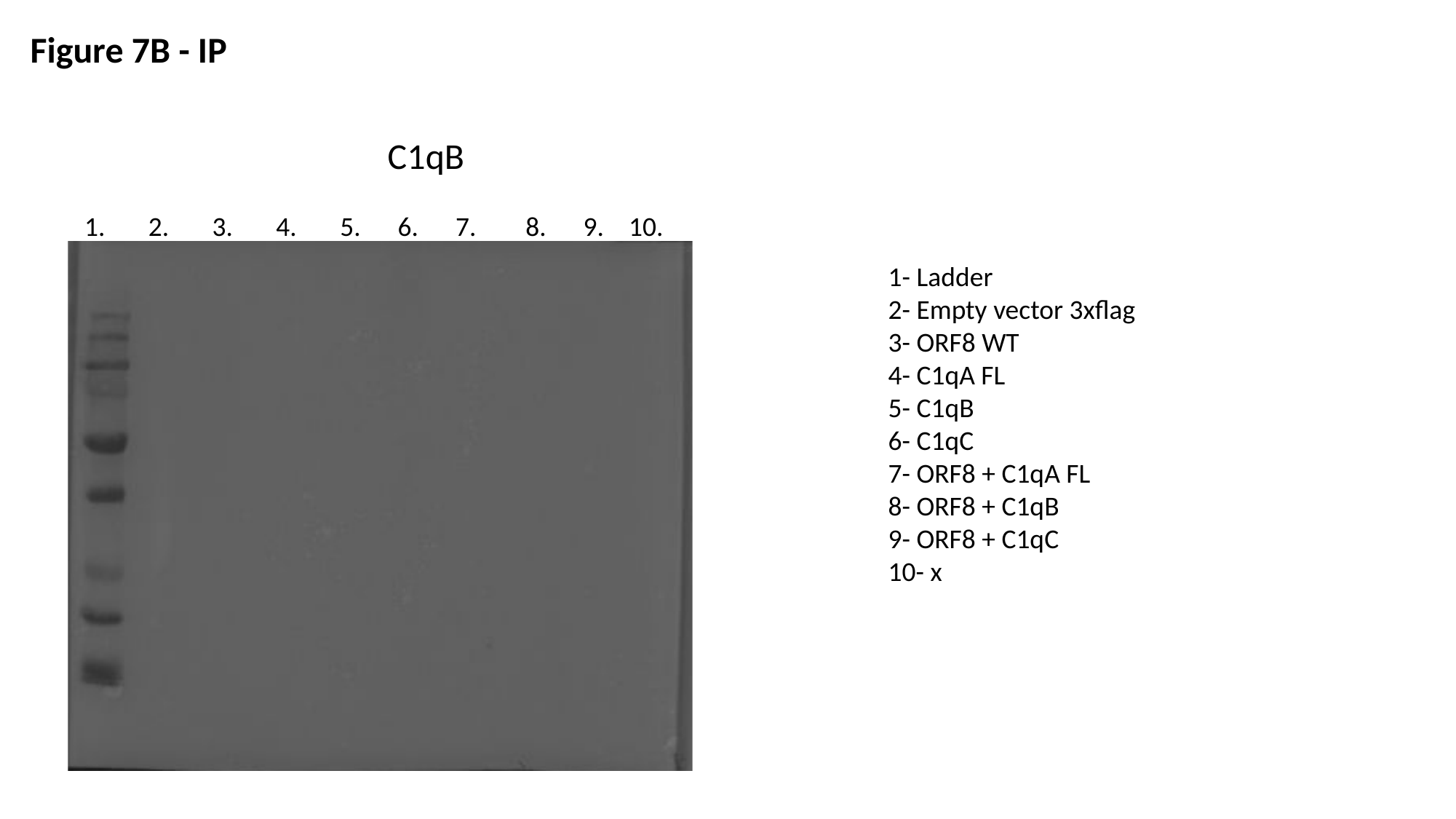

Figure 7B - IP
C1qB
1. 2. 3. 4. 5. 6. 7. 8. 9. 10.
1- Ladder
2- Empty vector 3xflag
3- ORF8 WT
4- C1qA FL
5- C1qB
6- C1qC
7- ORF8 + C1qA FL
8- ORF8 + C1qB
9- ORF8 + C1qC
10- x

## Slide 11
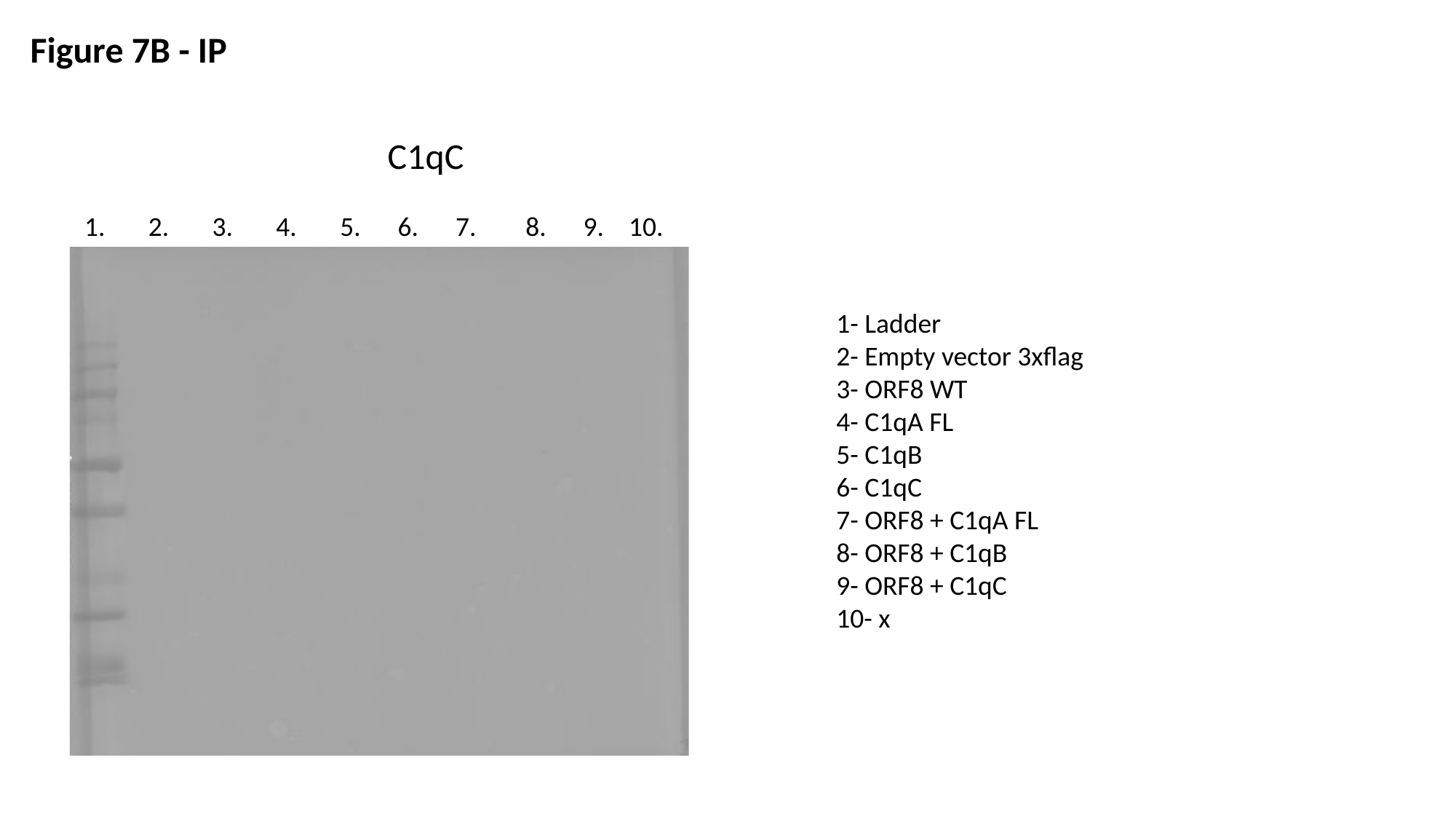

Figure 7B - IP
C1qC
1. 2. 3. 4. 5. 6. 7. 8. 9. 10.
1- Ladder
2- Empty vector 3xflag
3- ORF8 WT
4- C1qA FL
5- C1qB
6- C1qC
7- ORF8 + C1qA FL
8- ORF8 + C1qB
9- ORF8 + C1qC
10- x

## Slide 12
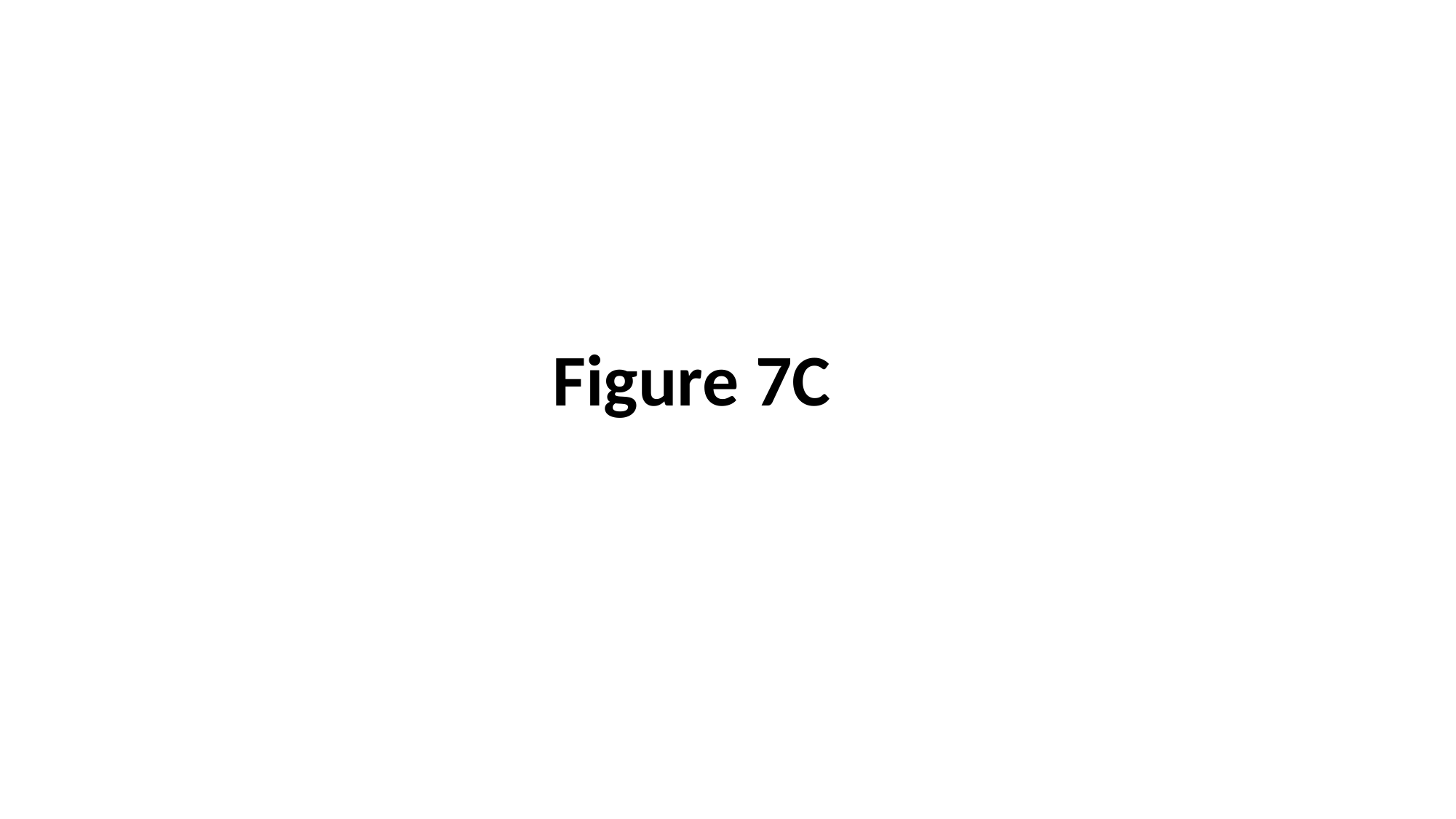

Figure 7C

## Slide 13
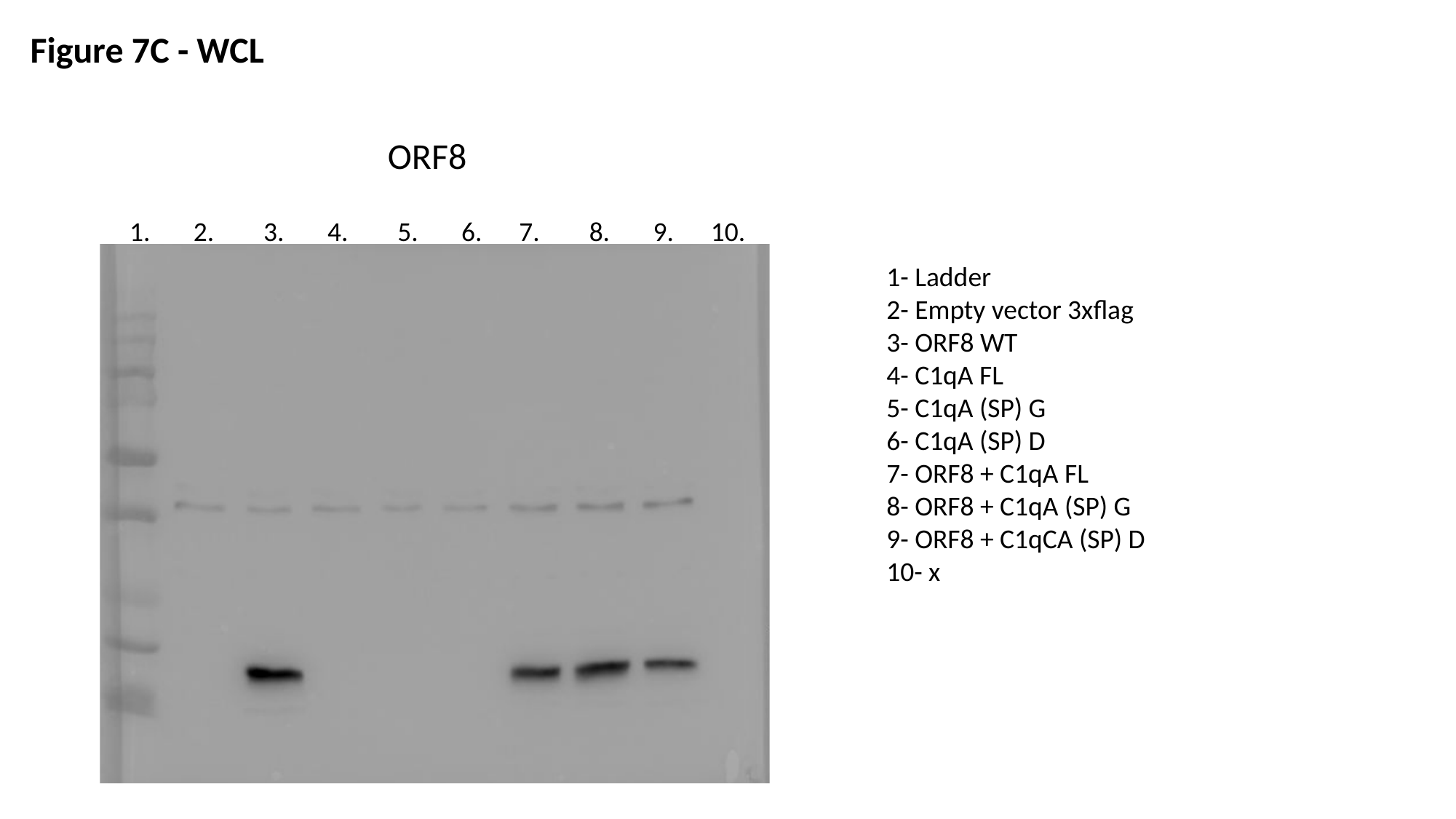

Figure 7C - WCL
ORF8
1. 2. 3. 4. 5. 6. 7. 8. 9. 10.
1- Ladder
2- Empty vector 3xflag
3- ORF8 WT
4- C1qA FL
5- C1qA (SP) G
6- C1qA (SP) D
7- ORF8 + C1qA FL
8- ORF8 + C1qA (SP) G
9- ORF8 + C1qCA (SP) D
10- x

## Slide 14
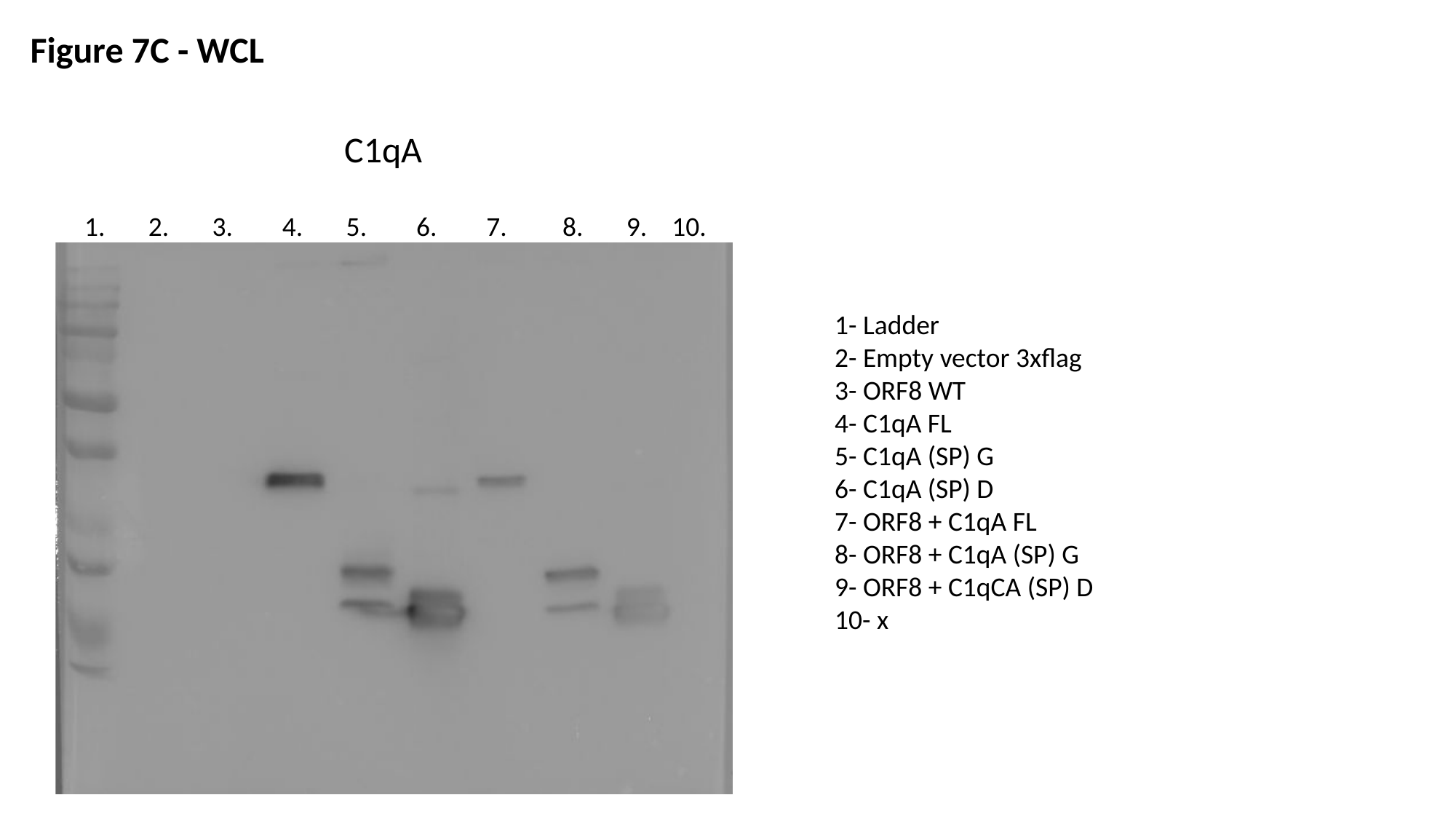

Figure 7C - WCL
C1qA
1. 2. 3. 4. 5. 6. 7. 8. 9. 10.
1- Ladder
2- Empty vector 3xflag
3- ORF8 WT
4- C1qA FL
5- C1qA (SP) G
6- C1qA (SP) D
7- ORF8 + C1qA FL
8- ORF8 + C1qA (SP) G
9- ORF8 + C1qCA (SP) D
10- x

## Slide 15
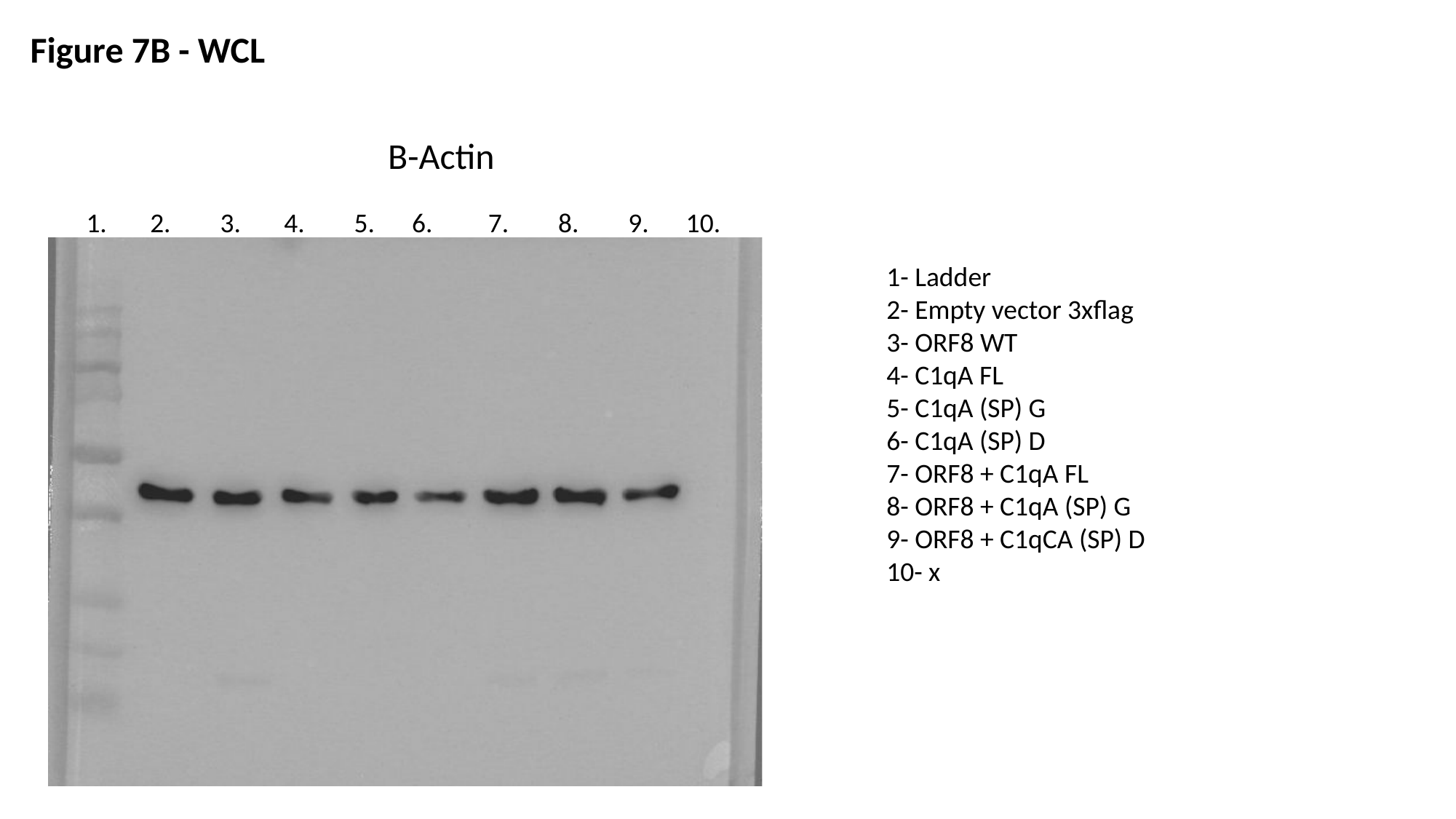

Figure 7B - WCL
B-Actin
1. 2. 3. 4. 5. 6. 7. 8. 9. 10.
1- Ladder
2- Empty vector 3xflag
3- ORF8 WT
4- C1qA FL
5- C1qA (SP) G
6- C1qA (SP) D
7- ORF8 + C1qA FL
8- ORF8 + C1qA (SP) G
9- ORF8 + C1qCA (SP) D
10- x

## Slide 16
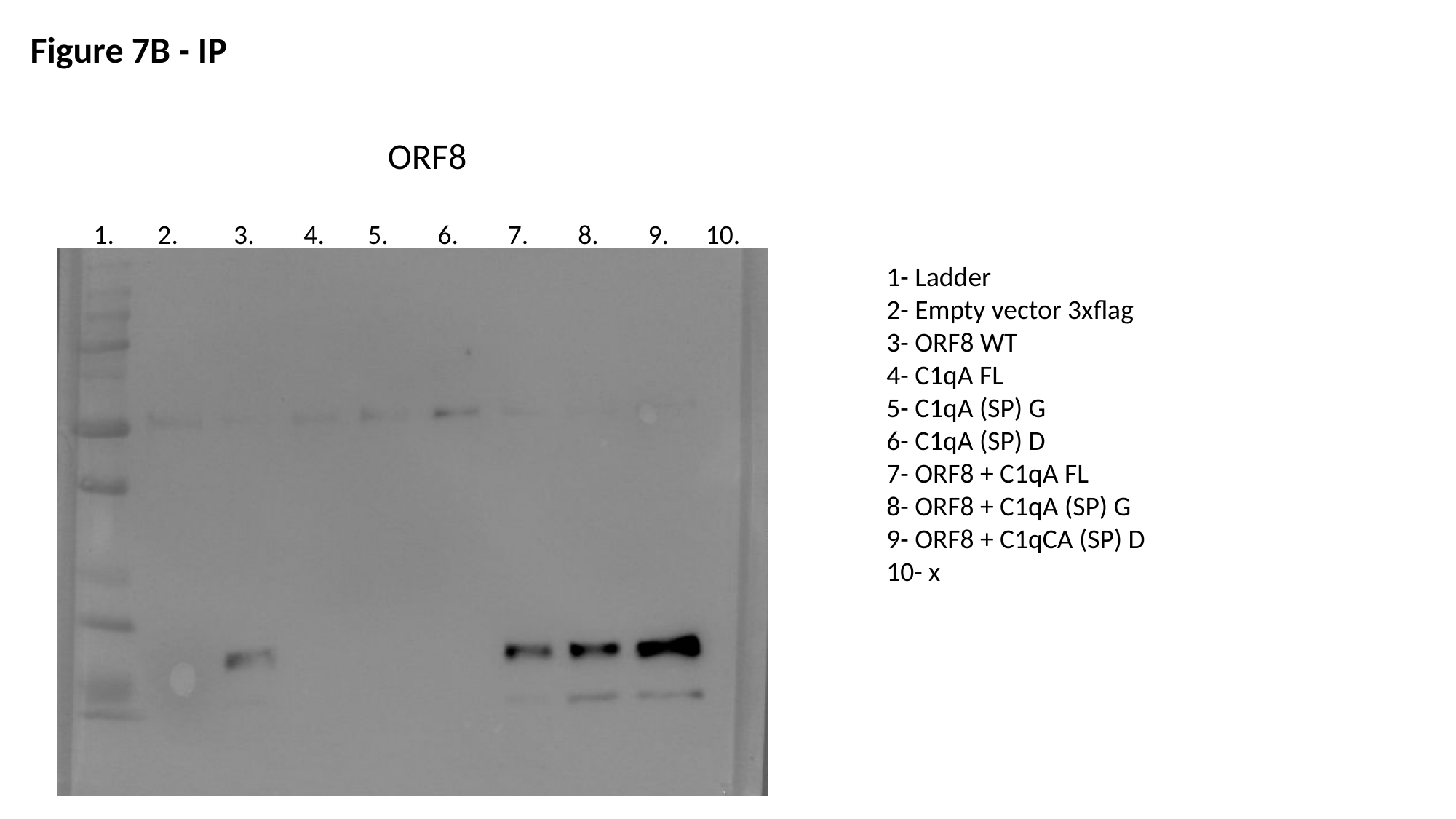

Figure 7B - IP
ORF8
1. 2. 3. 4. 5. 6. 7. 8. 9. 10.
1- Ladder
2- Empty vector 3xflag
3- ORF8 WT
4- C1qA FL
5- C1qA (SP) G
6- C1qA (SP) D
7- ORF8 + C1qA FL
8- ORF8 + C1qA (SP) G
9- ORF8 + C1qCA (SP) D
10- x

## Slide 17
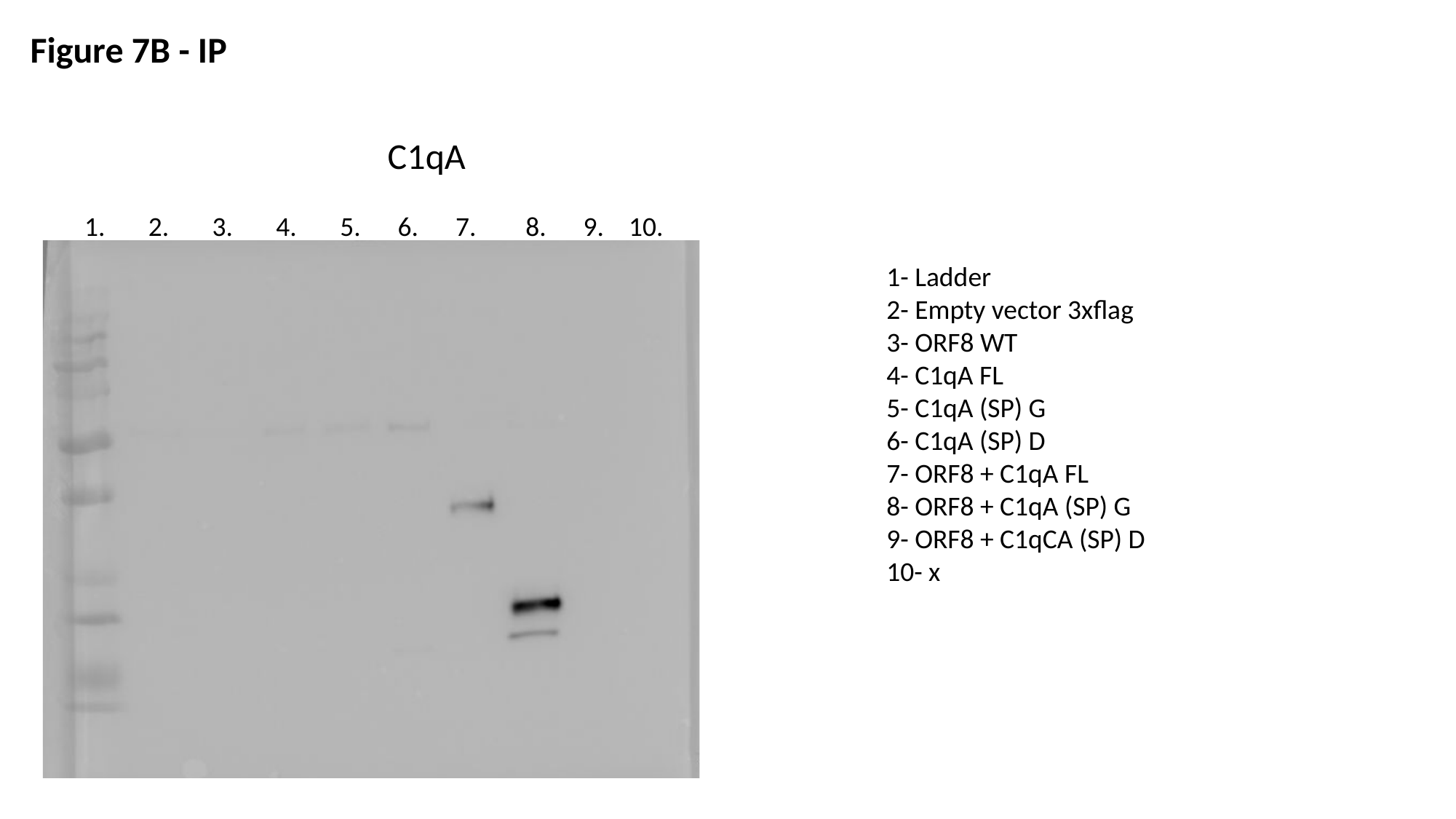

Figure 7B - IP
C1qA
1. 2. 3. 4. 5. 6. 7. 8. 9. 10.
1- Ladder
2- Empty vector 3xflag
3- ORF8 WT
4- C1qA FL
5- C1qA (SP) G
6- C1qA (SP) D
7- ORF8 + C1qA FL
8- ORF8 + C1qA (SP) G
9- ORF8 + C1qCA (SP) D
10- x
