# Supplementary material for: Transplacental SARS-CoV-2 protein ORF8 binds to complement C1q to trigger fetal inflammation
Source: EMBO J. 2024 Oct 10;43(22):10. doi: 10.1038/s44318-024-00260-9 (PMC11574245; doi:10.1038/s44318-024-00260-9)
Supplement: Supplementary file 14 — EV Figure Source Data [file 44318_2024_260_MOESM14_ESM.zip › Figure_EV_6.pptx]

## Slide 1
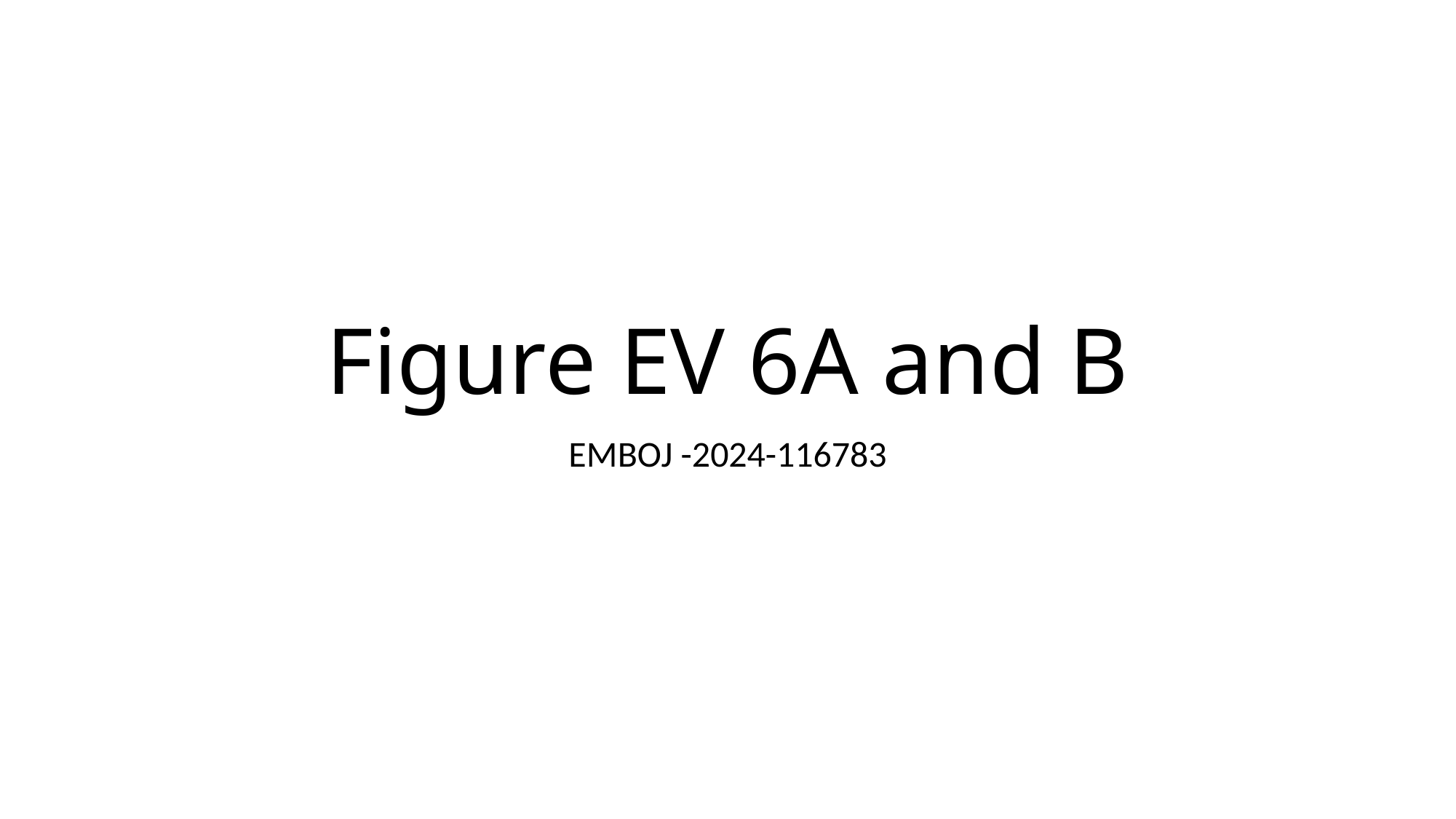

# Figure EV 6A and B
EMBOJ -2024-116783

## Slide 2
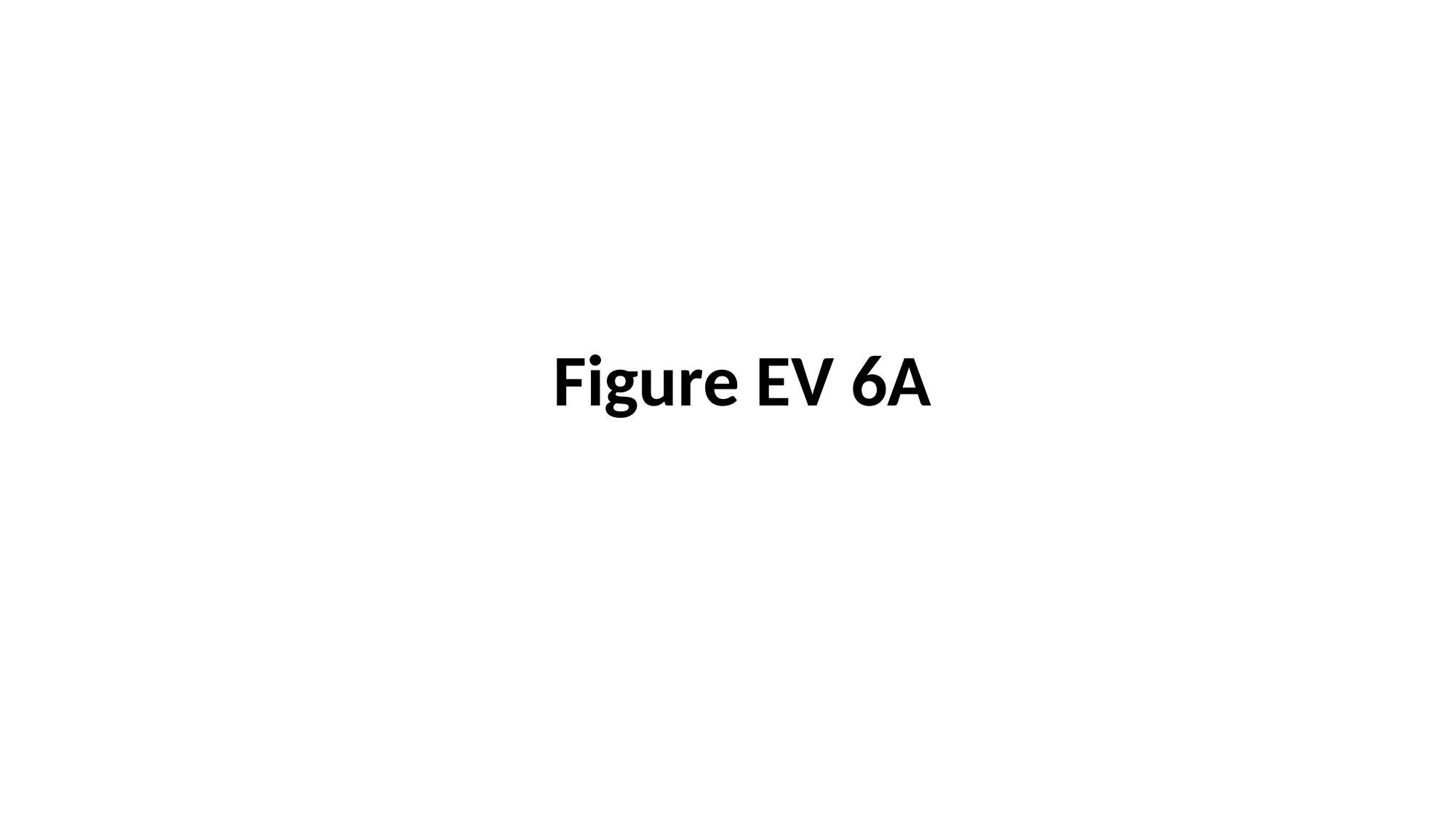

Figure EV 6A

## Slide 3
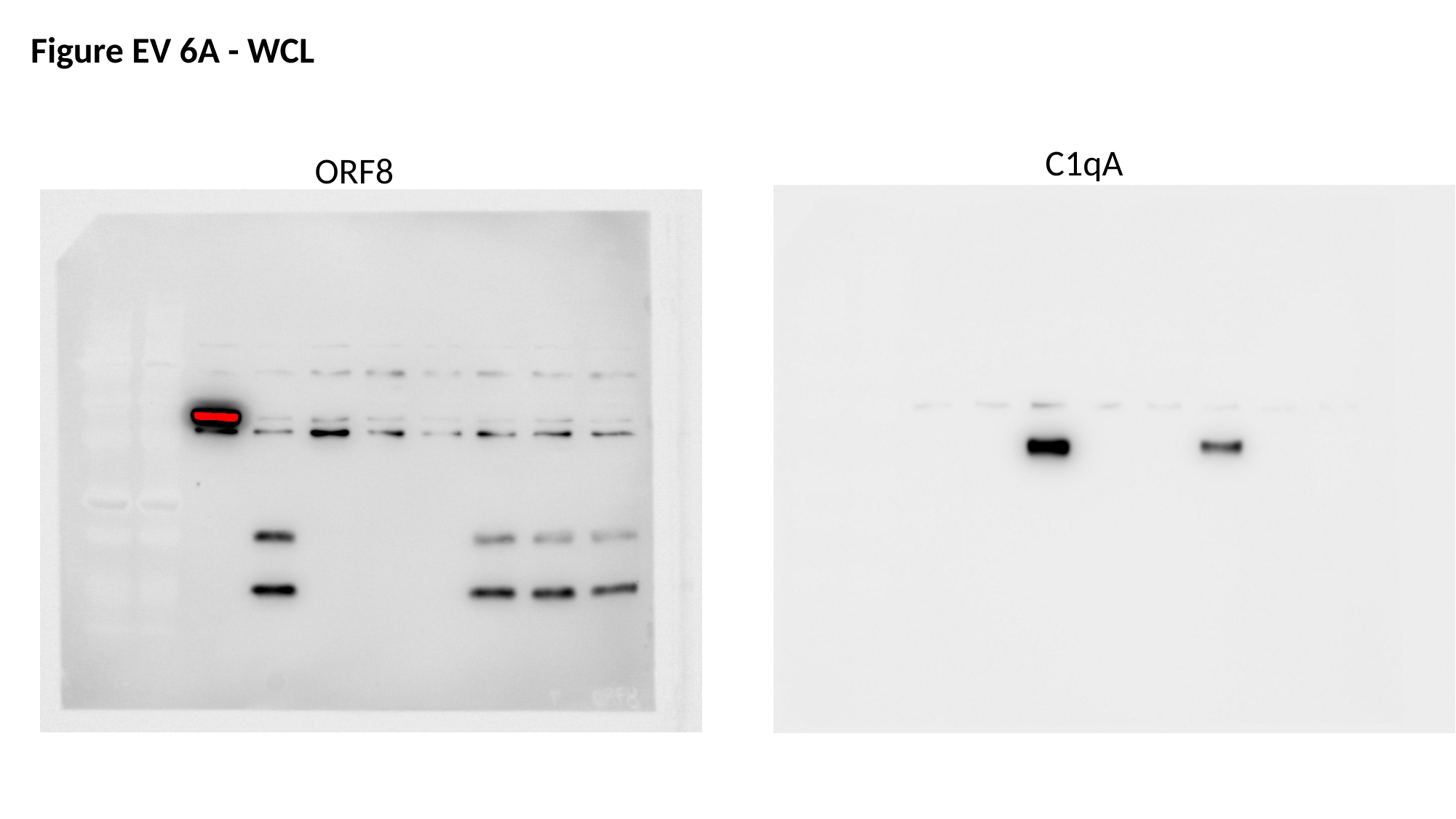

Figure EV 6A - WCL
C1qA
ORF8

## Slide 4
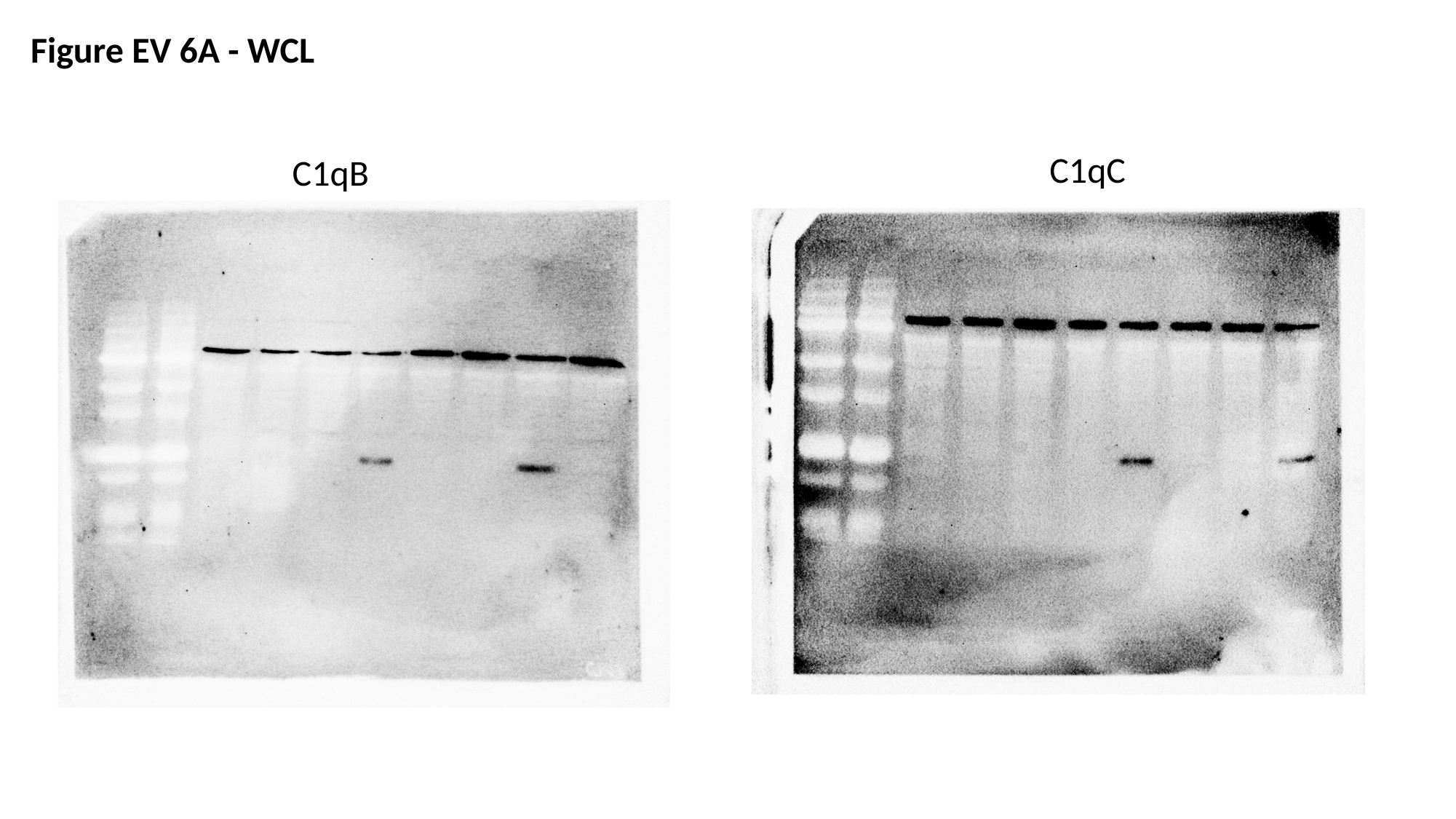

Figure EV 6A - WCL
C1qC
C1qB

## Slide 5
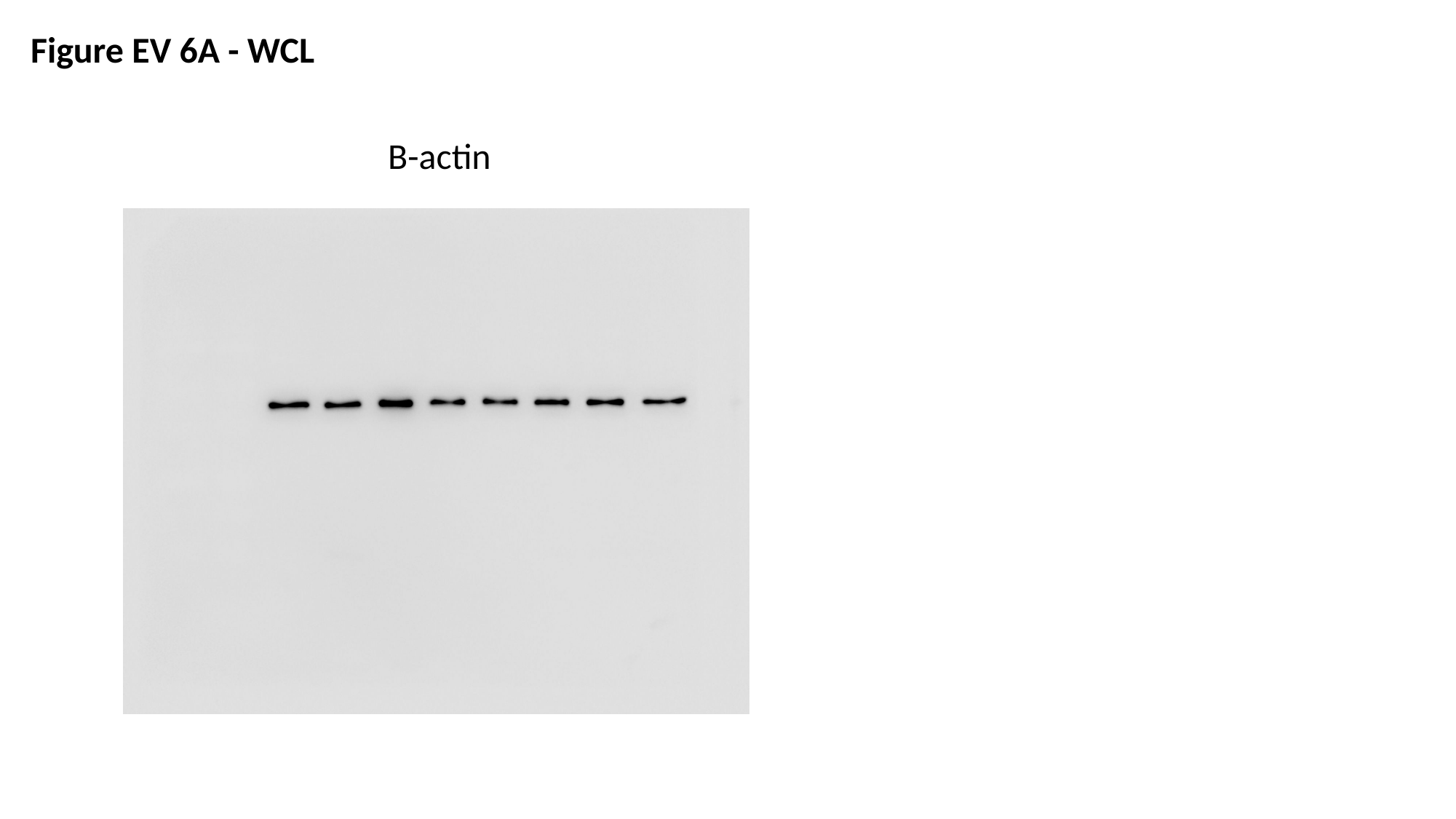

Figure EV 6A - WCL
B-actin

## Slide 6
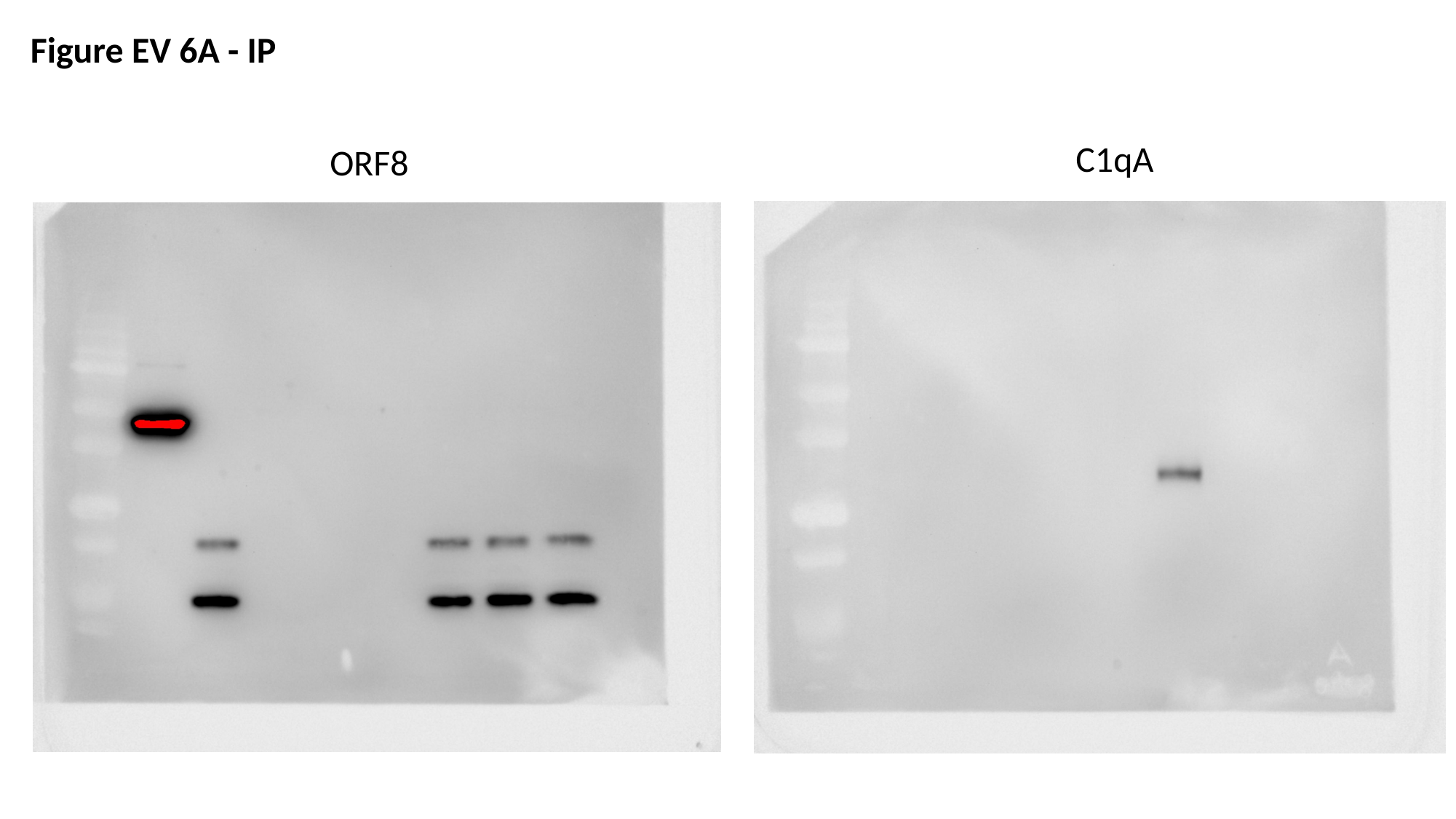

Figure EV 6A - IP
C1qA
ORF8

## Slide 7
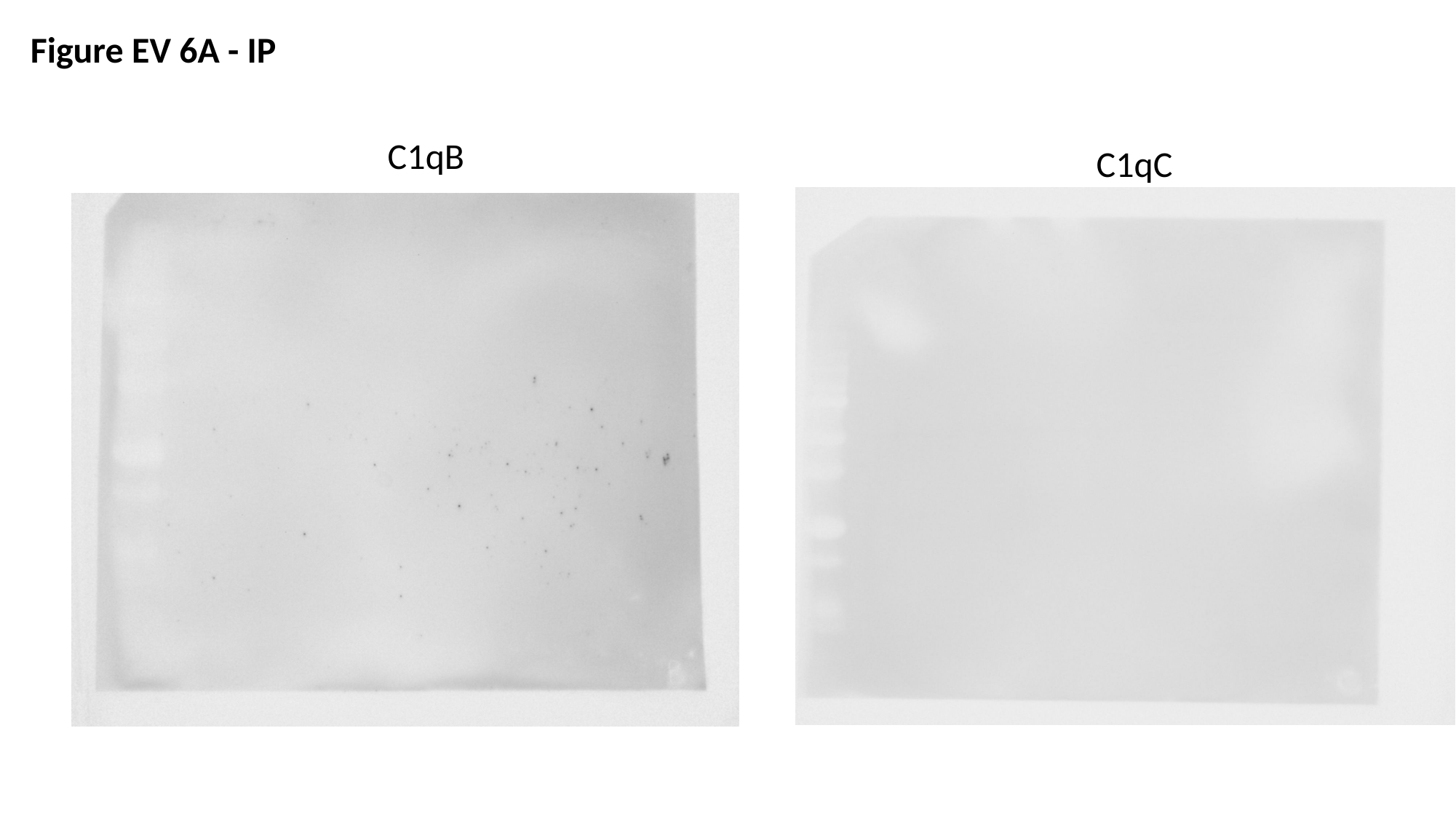

Figure EV 6A - IP
C1qB
C1qC

## Slide 8
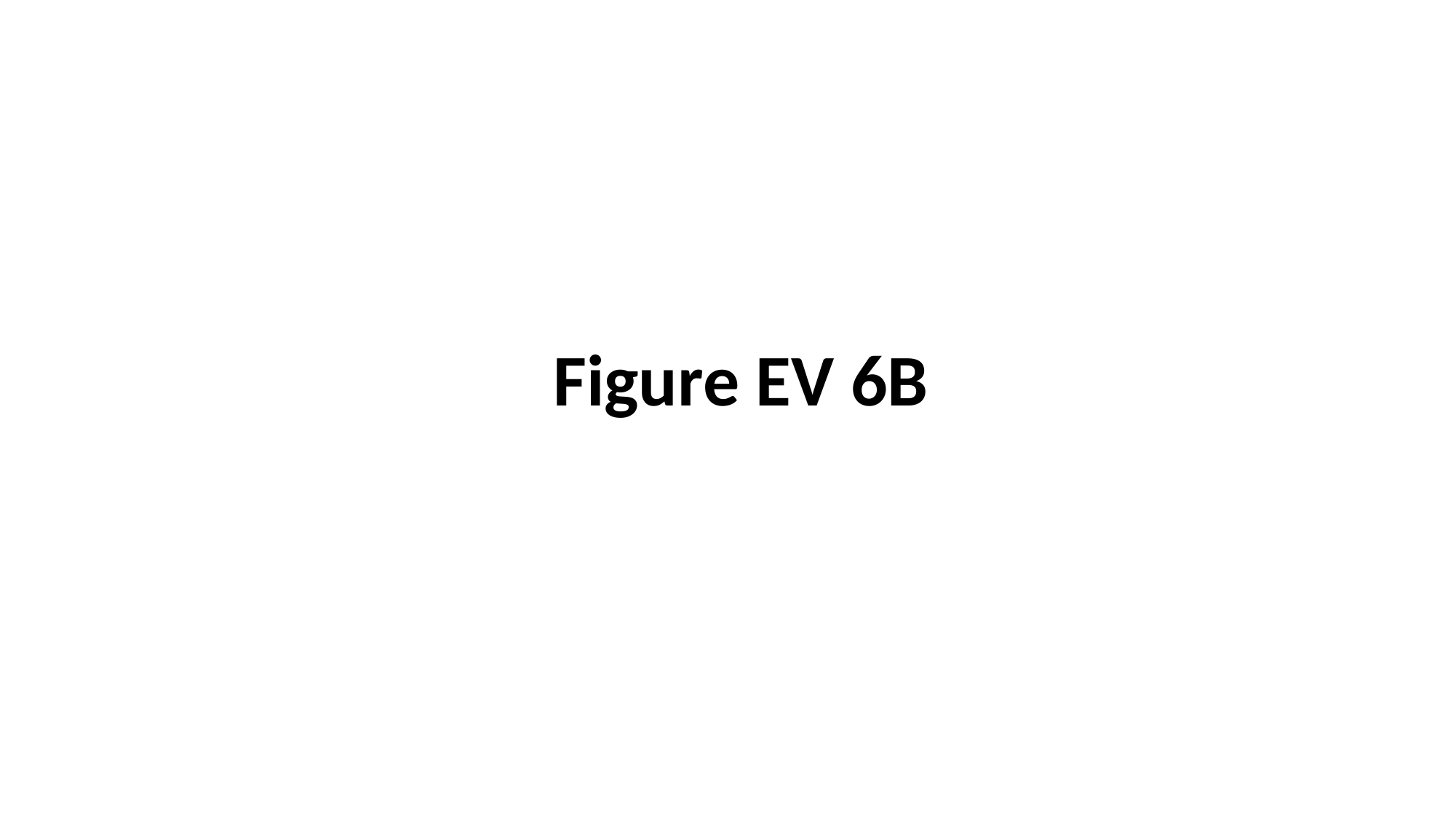

Figure EV 6B

## Slide 9
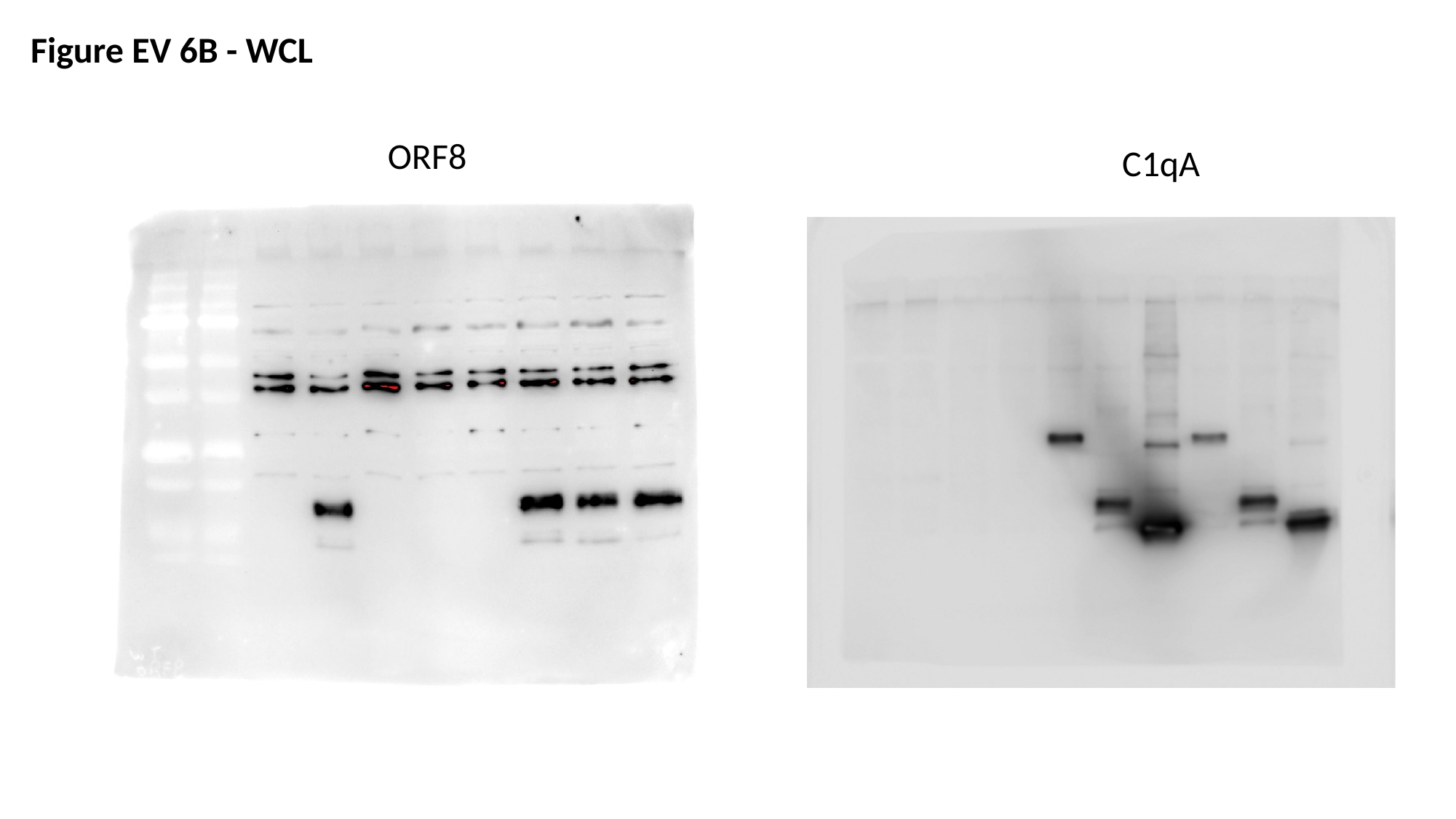

Figure EV 6B - WCL
ORF8
C1qA

## Slide 10
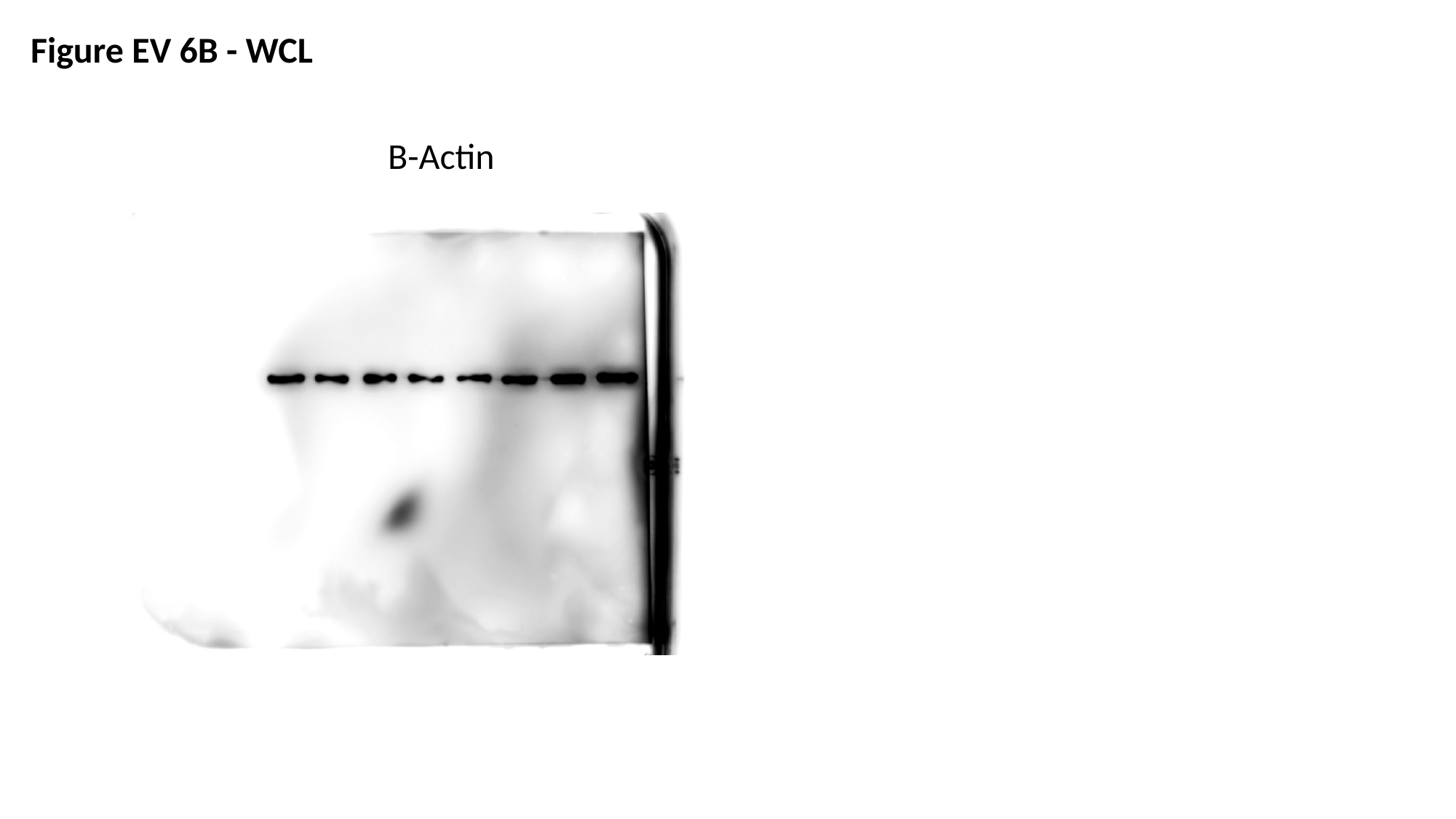

Figure EV 6B - WCL
B-Actin

## Slide 11
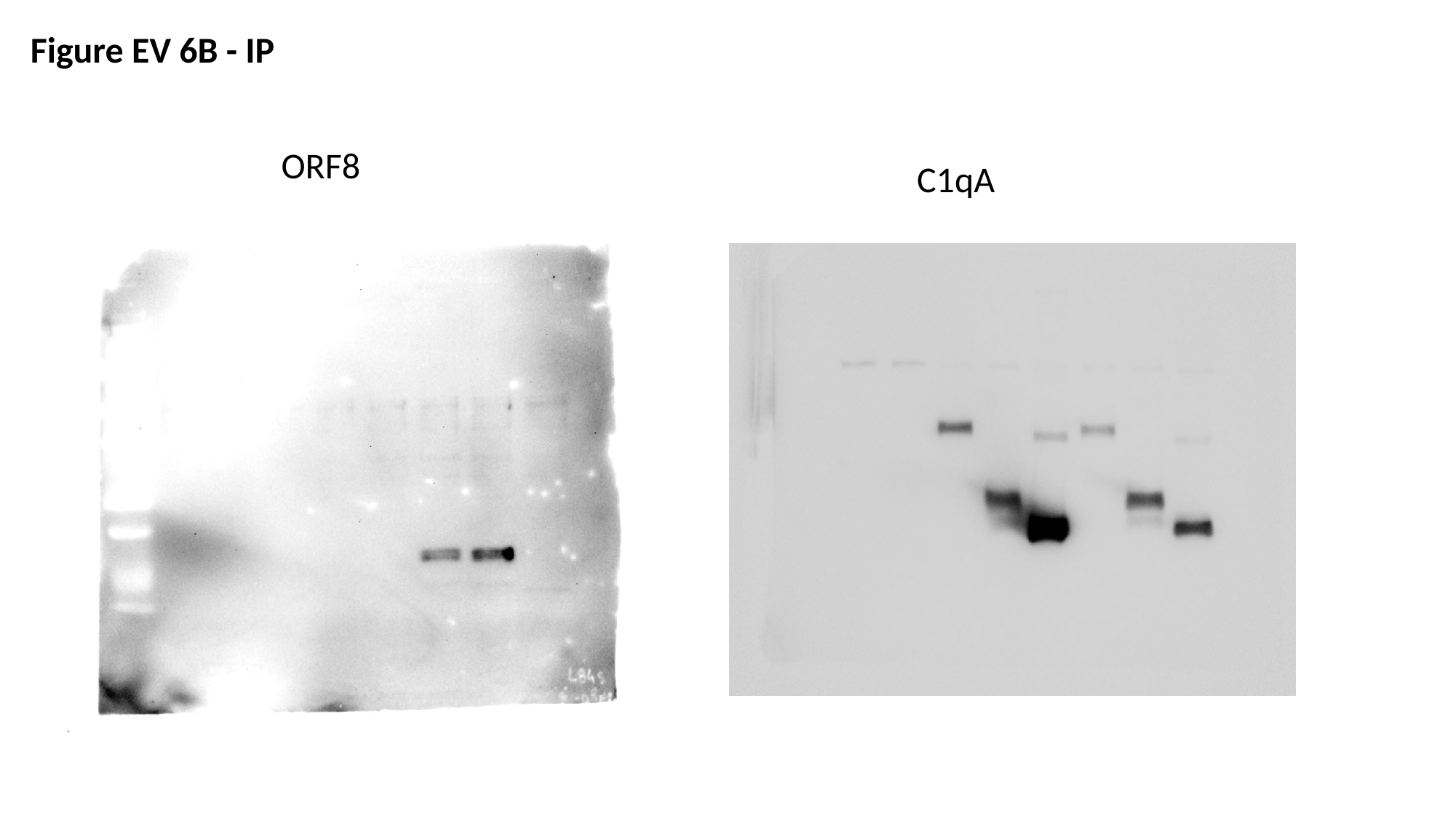

Figure EV 6B - IP
ORF8
C1qA
