# Supplementary material for: Transplacental SARS-CoV-2 protein ORF8 binds to complement C1q to trigger fetal inflammation
Source: EMBO J. 2024 Oct 10;43(22):10. doi: 10.1038/s44318-024-00260-9 (PMC11574245; doi:10.1038/s44318-024-00260-9)
Supplement: Supplementary file 15 — Expanded View Figures [file 44318_2024_260_MOESM15_ESM.pdf]

Expanded View Figures

Figure EV1. Complement-associated inflammatory responses in umbilical cord delivered from COVID-19-affected pregnancies. ▶

(**A**) tSNE plot of global transcriptomics for chorion of controls (emerald) and COVID-19 (purple). Volcano plot representing genes upregulated (red dots) and downregulated (blue dots) in the umbilical cord of COVID-19 relative to controls. (**B**) Bar plot representing gene ontology biological pathways associated with upregulated DEGs in the umbilical cord of COVID-19 relative to controls. (**C**) Violin plots representing the expression count values for individual genes related to complement activation in control ($n = 5$–6) and umbilical cords from COVID-19-affected pregnancies ($n = 19$). (**D**) Network analysis of DEGs in umbilical cords from COVID-19-affected pregnancies relative to controls. (**E**) Violin plots representing the expression count values for individual genes related to complement-associated inflammation in control ($n = 6$) and umbilical cords from COVID-19-affected pregnancies ($n = 19$). (**F**) Comparative analysis of DEGs ($-2 < FC > 2$, $p < 0.05$) in COVID-19 umbilical cord and amnion transcriptomics, depicting genes related to complement and complement-associated inflammation. Upregulated genes in red, downregulated genes in blue. Data are presented as means ± SEMs, using Mann–Whitney U test ($p < 0.05$). Source data are available online for this figure.

 © The Author(s)

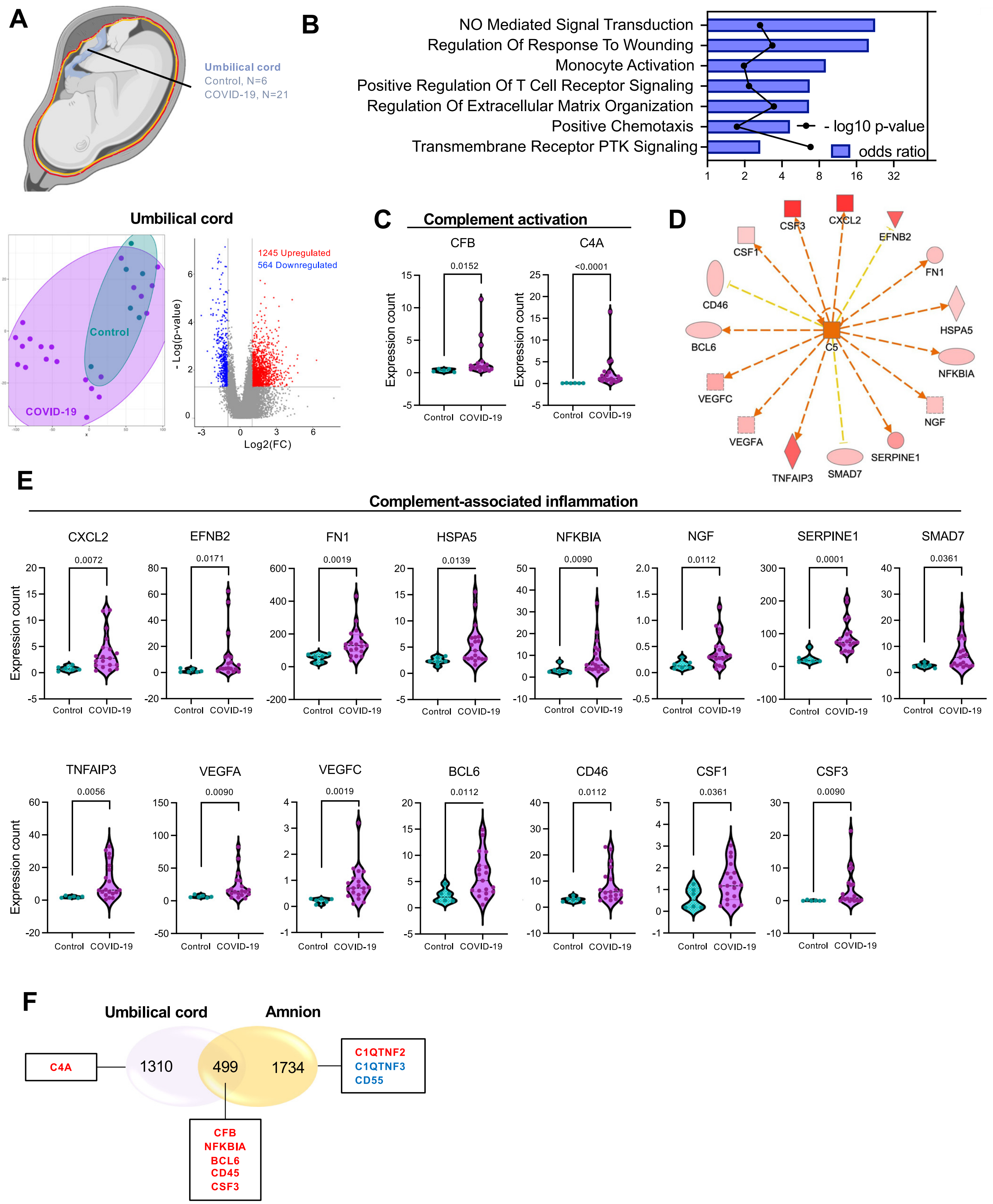
A
Umbilical cord
Control, N=6
COVID-19, N=21
B
NO Mediated Signal Transduction
Regulation Of Response To Wounding
Monocyte Activation
Positive Regulation Of T Cell Receptor Signaling
Regulation Of Extracellular Matrix Organization
Positive Chemotaxis
Transmembrane Receptor PTK Signaling
- log10 p-value
odds ratio
Umbilical cord
Control
COVID-19
1245 Upregulated
564 Downregulated
- Log(p-value)
Log2(FC)
C
Complement activation
CFB
C4A
0.0152
<0.0001
Expression count
Control COVID-19
D
CSF3 CXCL2 EFNB2 CSF1 FN1 CD46 HSPA5 BCL6 C5 NFKBIA VEGFC NGF VEGFA SERPINE1 TNFAIP3 SMAD7
E
Complement-associated inflammation
CXCL2 0.0072
EFNB2 0.0171
FN1 0.0019
HSPA5 0.0139
NFKBIA 0.0090
NGF 0.0112
SERPINE1 0.0001
SMAD7 0.0361
TNFAIP3 0.0056
VEGFA 0.0090
VEGFC 0.0019
BCL6 0.0112
CD46 0.0112
CSF1 0.0361
CSF3 0.0090
Expression count
Control COVID-19
F
Umbilical cord
Amnion
1310
499
1734
C4A
C1QTNF2
C1QTNF3
CD55
CFB
NFKBIA
BCL6
CD45
CSF3

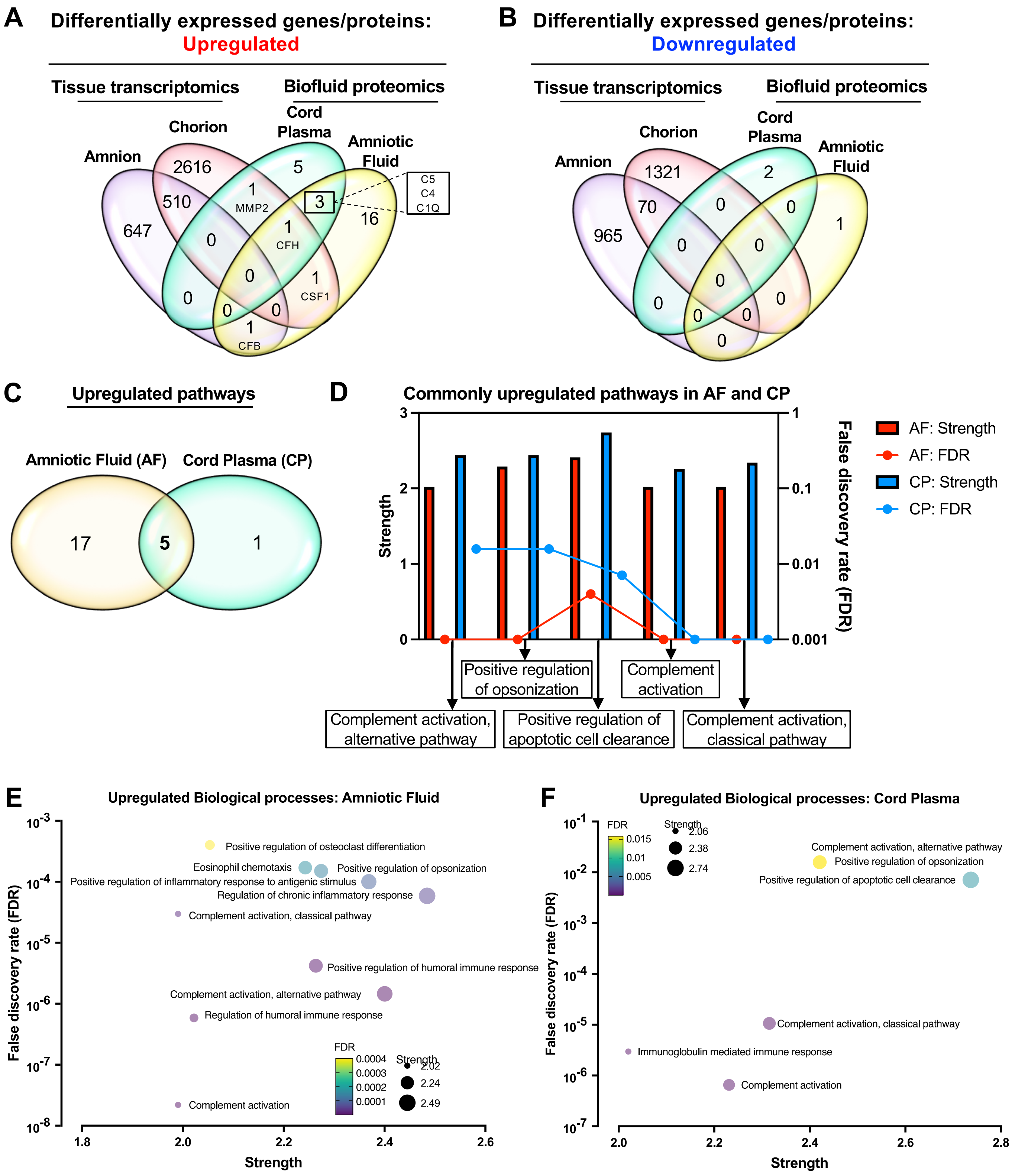
A
Differentially expressed genes/proteins:
Upregulated
Tissue transcriptomics
Biofluid proteomics
Amnion
Chorion
Cord Plasma
Amniotic Fluid
2616
510
647
5
1
MMP2
3
C5
C4
C1Q
16
1
CFH
1
CSF1
1
CFB
B
Differentially expressed genes/proteins:
Downregulated
Tissue transcriptomics
Biofluid proteomics
Amnion
Chorion
Cord Plasma
Amniotic Fluid
1321
70
965
2
1
C
Upregulated pathways
Amniotic Fluid (AF)
Cord Plasma (CP)
17
5
1
D
Commonly upregulated pathways in AF and CP
Strength
False discovery rate (FDR)
AF: Strength
AF: FDR
CP: Strength
CP: FDR
Positive regulation of opsonization
Complement activation
Complement activation, alternative pathway
Positive regulation of apoptotic cell clearance
Complement activation, classical pathway
E
Upregulated Biological processes: Amniotic Fluid
Positive regulation of osteoclast differentiation
Eosinophil chemotaxis
Positive regulation of opsonization
Positive regulation of inflammatory response to antigenic stimulus
Regulation of chronic inflammatory response
Complement activation, classical pathway
Positive regulation of humoral immune response
Complement activation, alternative pathway
Regulation of humoral immune response
Complement activation
False discovery rate (FDR)
Strength
FDR
F
Upregulated Biological processes: Cord Plasma
Complement activation, alternative pathway
Positive regulation of opsonization
Positive regulation of apoptotic cell clearance
Complement activation, classical pathway
Immunoglobulin mediated immune response
Complement activation
False discovery rate (FDR)
Strength
FDR

 © The Author(s)

Figure EV2. Comparison analysis of placental tissue transcriptomics and biofluids proteomics derived from COVID19-affected pregnancies at delivery.

Graphical representation of (**A**) upregulated and (**B**) downregulated differentially expressed genes/proteins in tissue transcriptomics (Chorion, $n = 21$ and; Amnion, $n = 18$) and biofluid proteomics (Amniotic fluid, $n = 8$; and Cord plasma, $n = 20$) from COVID-19 affected pregnancies. (**C**) Comparative analysis of upregulated genes/proteins in amniotic fluid and cord plasma from COVID-19-affected pregnancies. (**D**) Commonly upregulated pathways in amniotic fluid and cord plasma from COVID-19-affected pregnancies. (**E**, **F**) Bubble plots representing upregulated biological process in (**E**) Amniotic fluid and (**F**) cord plasma. Strength and False discovery rate (FDR) were calculated using STRING database pathway analysis. Source data are available online for this figure.

© The Author(s)

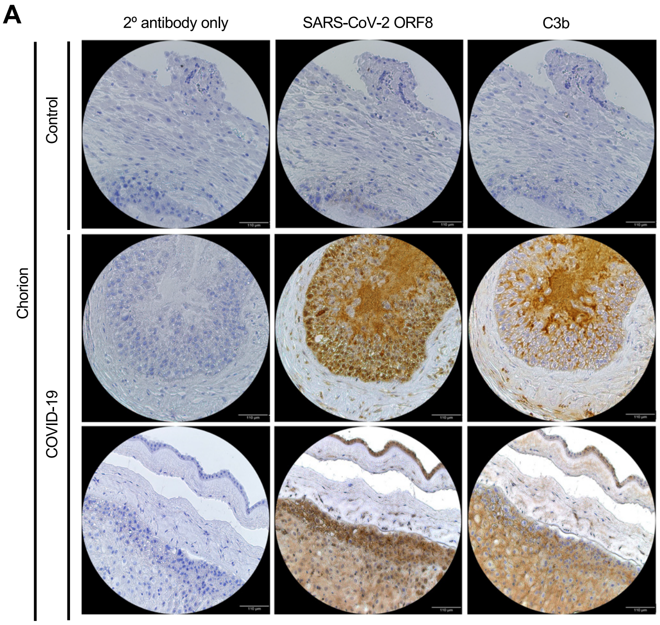

Figure EV3. Chorion tissues from COVID-19-affected pregnancies exhibited augmented complement activation at ORF8-positive sites.

(**A**) Chorion serial sections were analyzed by immunohistochemistry for detection of SARS-CoV-2 ORF8 and C3b. From left to right, tissues stained with (i) Hematoxylin (purple) and the secondary antibody anti-rabbit (Motulsky and Brown, 2006); (ii) Hematoxylin (purple) and anti-ORF8 produced (Motulsky and Brown, 2006); and (iii) Hematoxylin (purple) and anti-C3b (Motulsky and Brown, 2006). Images were taken at 20X magnification. Representative images from two control and two COVID-19 chorion specimens. Source data are available online for this figure.

 © The Author(s)

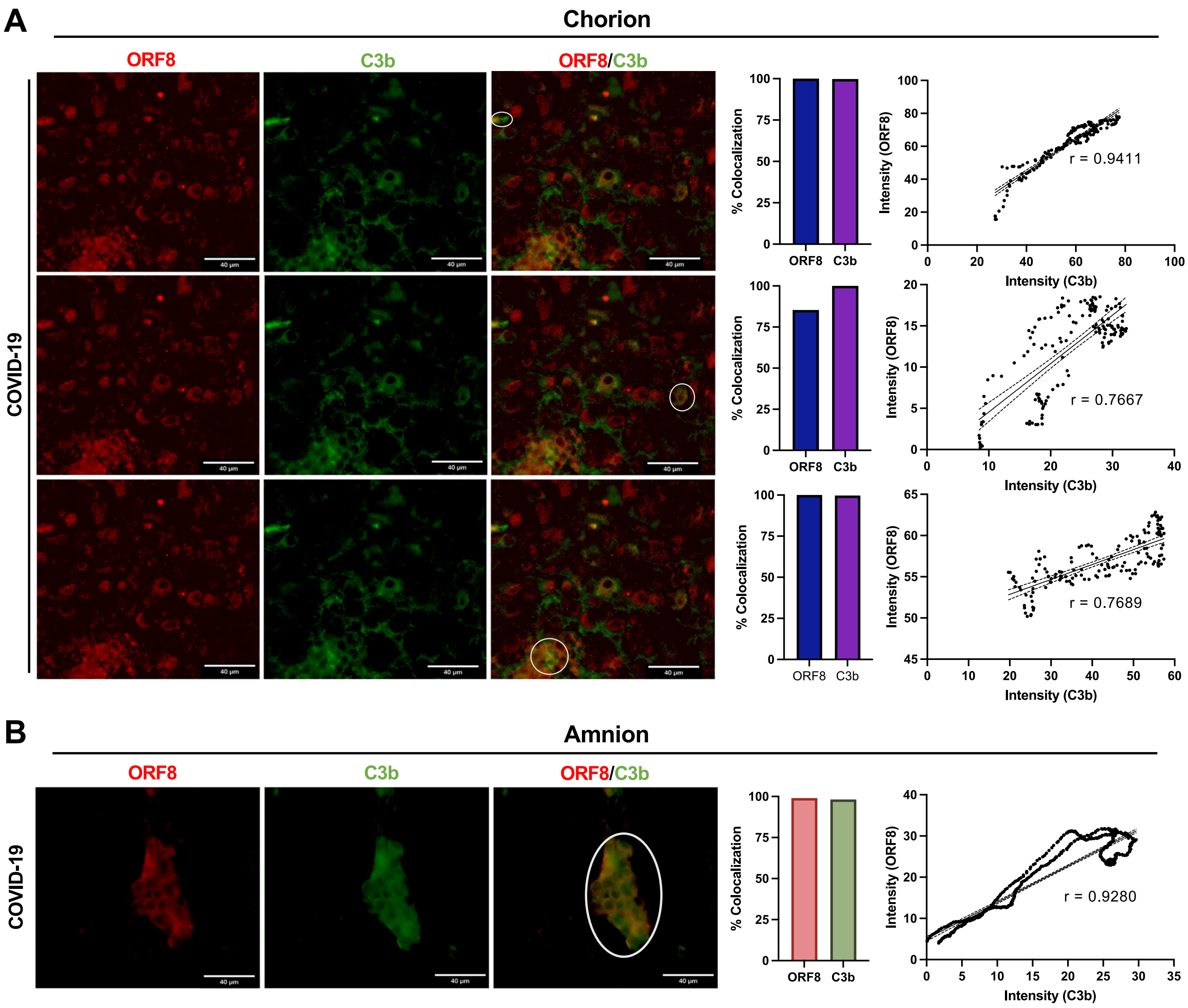

Figure EV4. Confirmed colocalization of ORF8 and C3b in chorion and amnion tissues from COVID-19-affected pregnancies by fluorescence signals.

(**A**) Chorion and (**B**) amnion serial sections were stained with SARS-CoV-2 ORF8 (red) and C3b (green). Mander's and Pearson's correlations coefficient between SARS-CoV-2 ORF8 and C3b. White circles represent the colocalization areas analyzed. Images were taken at 40x magnification. Images are representative of one COVID-19+ pregnancy. Scale bars: 40 μm. Source data are available online for this figure.

© The Author(s)

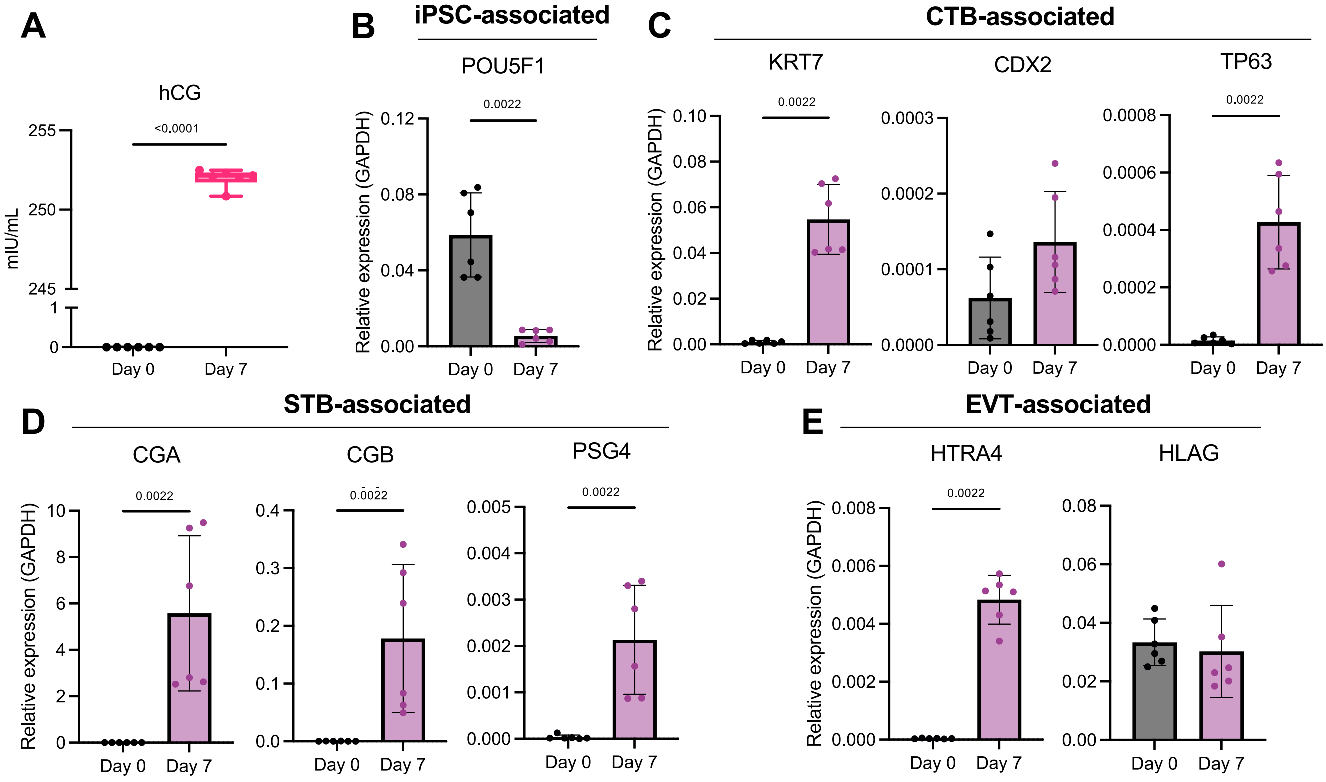

Figure EV5. Phenotypic characterization of trophoblast delivered from iPSCs.

(**A**) Boxplot showing the levels of human chorionic gonadotropin (hCG) in the supernatant of iPSC-derived trophoblasts cultures at day 0 and day 7 of differentiation. Data are presented in boxplot, middle line represents means, the bound of box represent interquartile range, and whiskers represent maximum and minimum values in mUI/mL from six independent experiments ($n = 6$). (**B–E**) Bar plots representing expression of genes associated as phenotypic markers in the iPSC-derived trophoblasts cultures ($n = 6$) at day 0 and day 7 of differentiation. (**B**) iPSC-associated transcripts—*POU5F1*, (**C**) CTB-associated transcripts—*KRT7*, *CDX2*, and *TP63*, (**D**) STB-associated transcripts—*CGA*, *CGB*, and *PSG4*, and (**E**) EVT-associated transcripts—*HTRA4*, and *HLAG*. Data are presented in scatter dot plot, line represent means, and whiskers standard derivation. Relative expression was calculated by DCt method using *GAPDH* as the normalizing gene. Data are presented as means ± SDs. Immortalized pluripotent stem cells (iPSC). Cytotrophoblast (CTB). Syncytiotrophoblasts (STB). Extravillous trophoblasts (EVT). POU Domain, Class 5, Transcription Factor 1 (*POU5F1*). Kerantin 7 (*KRT7*). Caudal Type Homeobox 2 (*CDX2*), Tumor Protein P63 (*TP63*). Glycoprotein Hormones, Alpha Polypeptide (*CGA*). Chorionic Gonadotropin Subunit Beta (*CGB*). Pregnancy Specific Beta-1-Glycoprotein 4 (*PSG4*). High-Temperature Requirement Factor A4 (*HTRA4*). Major Histocompatibility Complex, Class I, G (*HLAG*). Mann–Whitney U test was used for all analysis ($p < 0.05$). Source data are available online for this figure.

 © The Author(s)

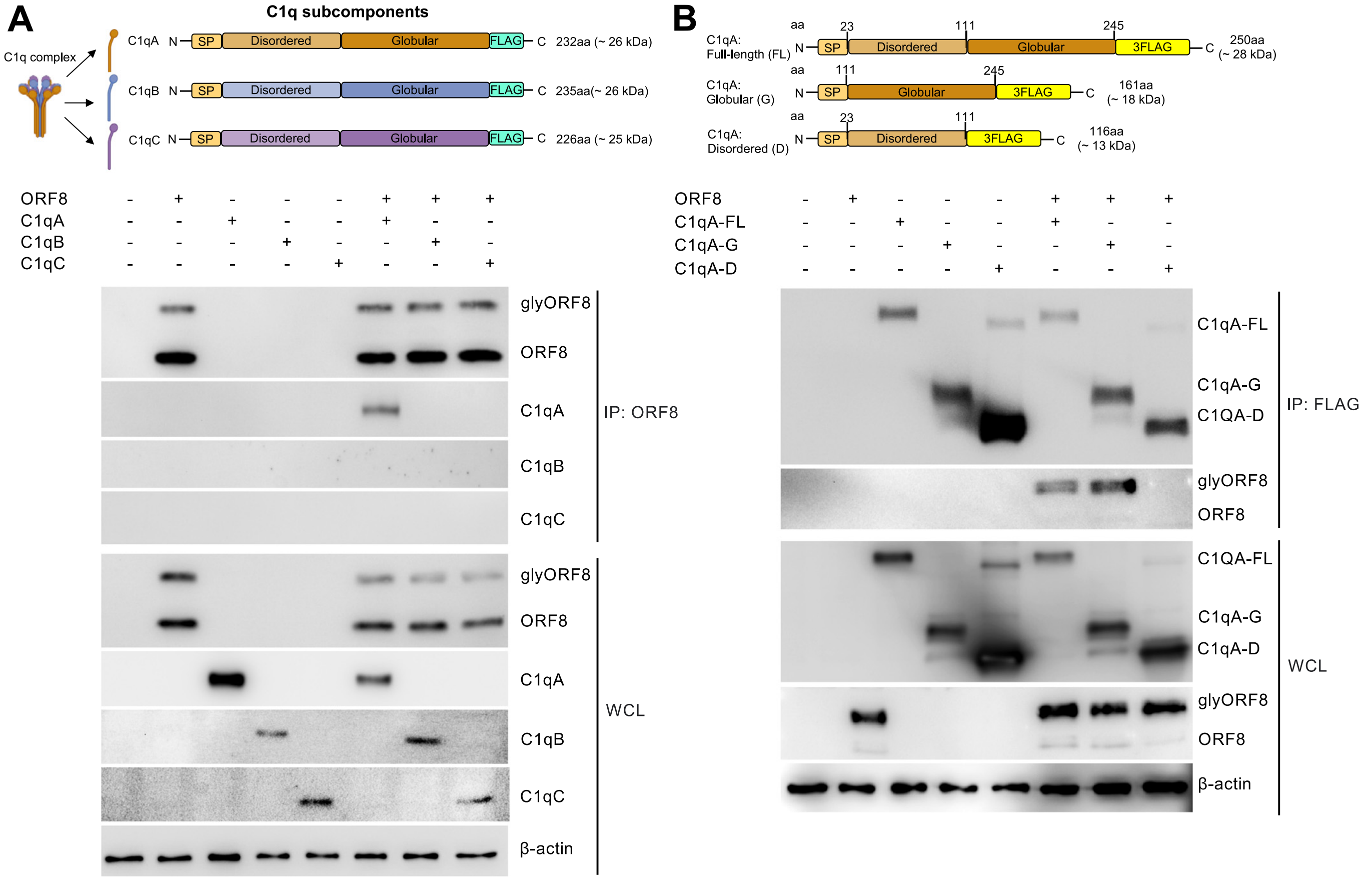

Figure EV6. SARS-CoV-2 ORF8 binds to globular domain of complement C1q subcomponent subunit A in HEK 293T cells.

(**A**) Co-immunoprecipitation of SARS-CoV-2 ORF8 with C1qA, C1qB, and C1qC. Graphical representation of the plasmid constructs containing the expression cassettes for the subcomponents C1qA, C1qB, and C1qC contained an N-terminal signal peptide (SP), and a C-terminal FLAG tag. Western blot images for co-immunoprecipitation using an anti-ORF8 antibody. (**B**) Co-immunoprecipitation of C1qA full-length (FL), globular (G), disordered domain (D) with SARS-CoV-2 ORF8. Graphical representation of the plasmid constructs containing expression cassettes for the C1qA-FL, C1qA-G, and C1qA-D, with N-terminal SP and a C-terminal 3FLAG tag, cloned in pIRES vectors. Western blot images for co-immunoprecipitation using an anti-Flag antibody. Co-immunoprecipitated product (IP). Whole-cell lysate (WCL). Source data are available online for this figure.

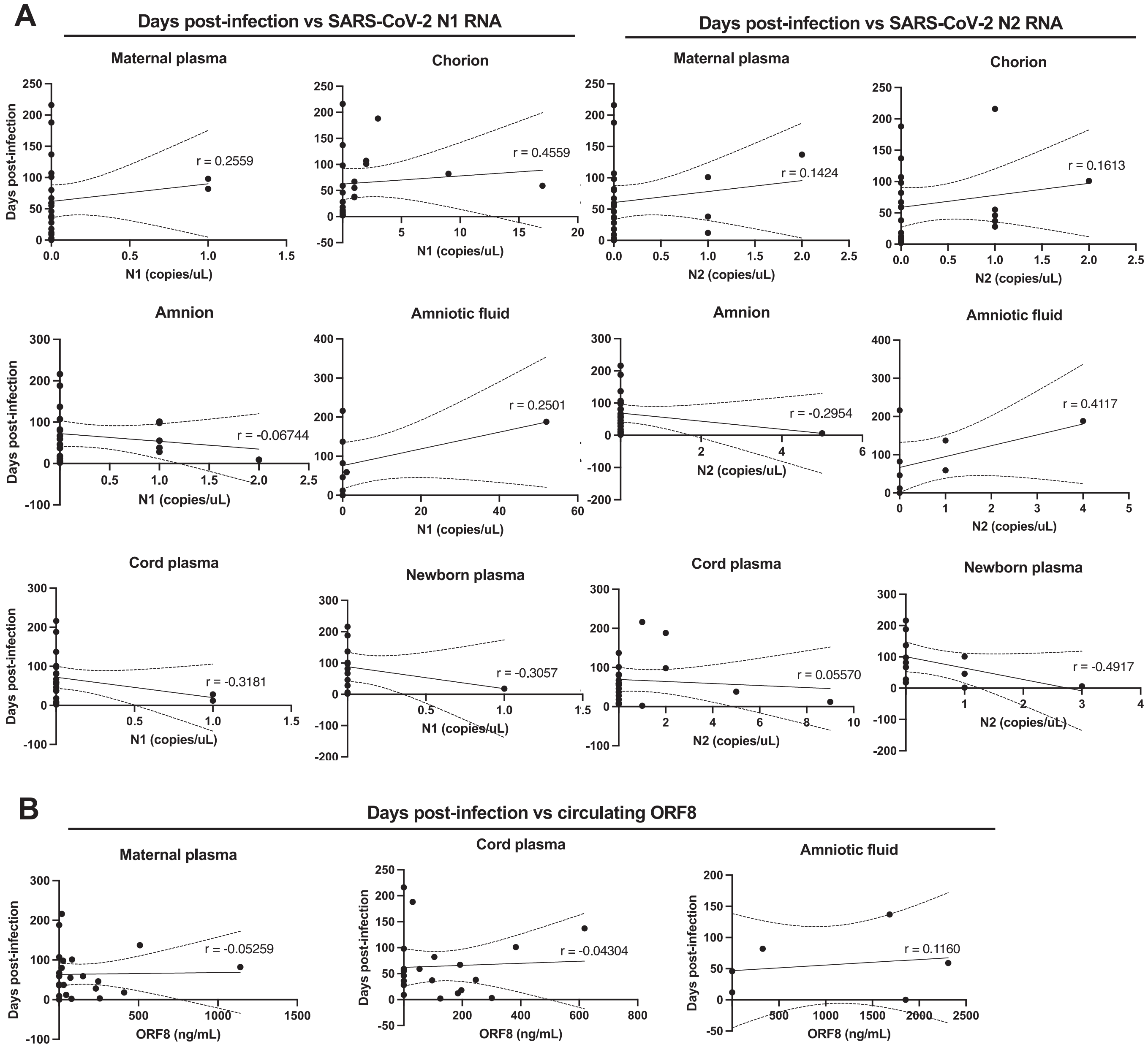

Figure EV7. Correlation analyses between the number of days post-infection to delivery and levels of SARS-CoV-2 N1/N2 RNA or circulating ORF8 in COVID-19 pregnancies.

Correlation analyses between the number of days post SARS-CoV-2 infection to delivery and (**A**) SARS-CoV-2 N1 RNA copies, SARS-CoV-2 N2 RNA copies, and (**B**) circulating ORF8 levels in all biospecimens analyzed, including maternal plasma, chorion, amnion, cord plasma and newborn plasma. Simples linear regression and Spearman's rank correlation test was used for all the correlations analysis. Scatter plots with Pearson correlation coefficients (r) and dotted lines represent 95% confidence intervals. Source data are available online for this figure.

 © The Author(s)

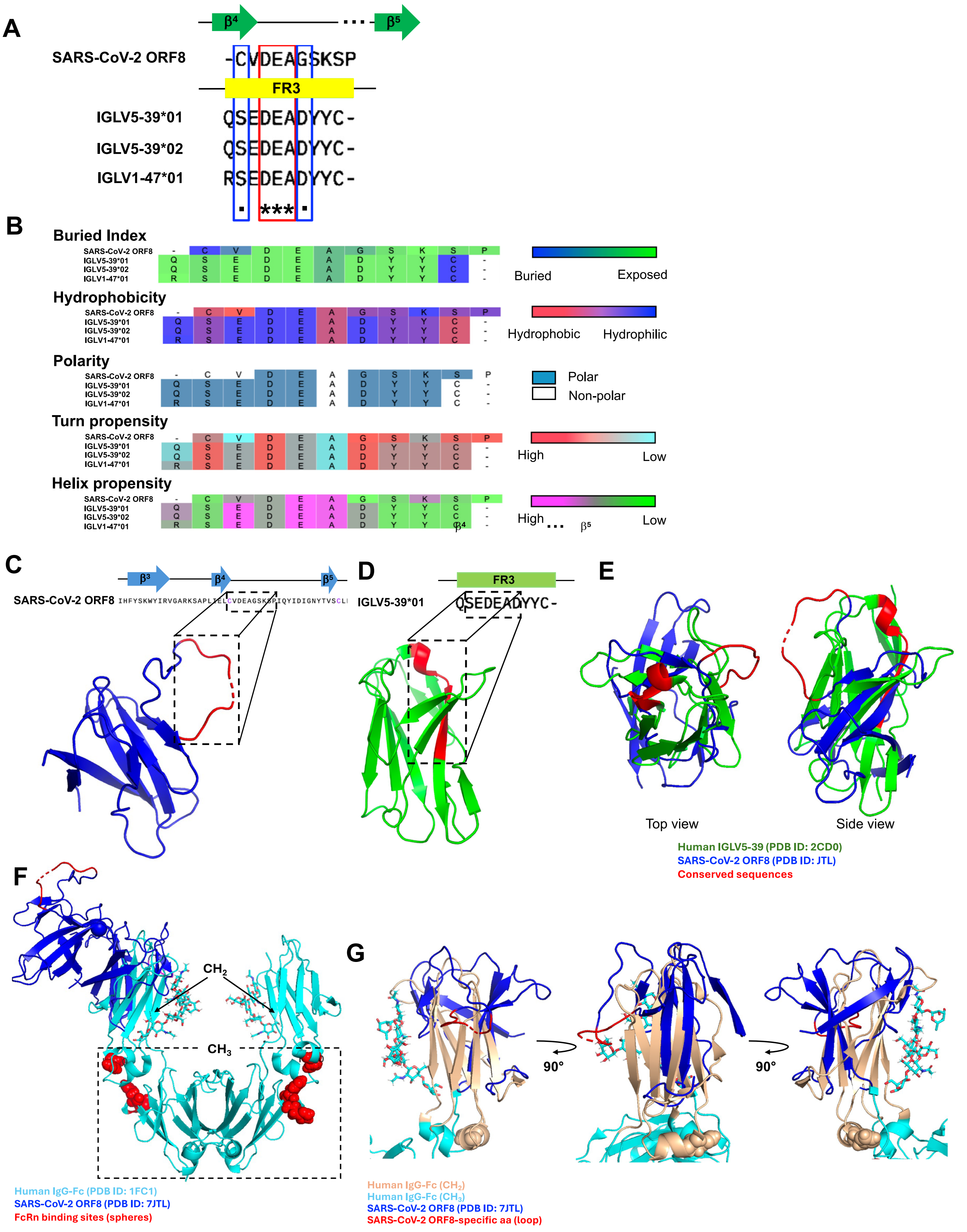
A
β4
β5
SARS-CoV-2 ORF8
-CVDEAGSKSP
FR3
IGLV5-39*01
QSEDEADYYC-
IGLV5-39*02
QSEDEADYYC-
IGLV1-47*01
RSEDEADYYC-
B
Buried Index
Buried
Exposed
Hydrophobicity
Hydrophobic
Hydrophilic
Polarity
Polar
Non-polar
Turn propensity
High
Low
Helix propensity
High
Low
C
β3
β4
β5
SARS-CoV-2 ORF8
D
FR3
IGLV5-39*01
QSEDEADYYC-
E
Top view
Side view
Human IGLV5-39 (PDB ID: 2CD0)
SARS-CoV-2 ORF8 (PDB ID: JTL)
Conserved sequences
F
CH2
CH3
Human IgG-Fc (PDB ID: 1FC1)
SARS-CoV-2 ORF8 (PDB ID: 7JTL)
FcRn binding sites (spheres)
G
90°
90°
Human IgG-Fc (CH2)
Human IgG-Fc (CH3)
SARS-CoV-2 ORF8 (PDB ID: 7JTL)
SARS-CoV-2 ORF8-specific aa (loop)

Figure EV8. Structural and sequence analysis of SARS-CoV-2 ORF8 and human immunoglobulin genes reveals conservation and functional insights.

(**A**) Sequence alignment of SARS-CoV-2 ORF8 and human immunoglobulin variable lambda chain (IGLV) genes. Representative amino acid residues of SARS-CoV-2 ORF8-specific region located at the loop flanking β^{4-} β^{5} strands were compared with a portion of the framework 3 (FR3) region of the variable light chain of human IGLV5-39*01, IGLV5-39*02, and IGLV1-47*01 germline gene sequences. Identical residues were highlighted in red box and marked by an asterisk (*), while semi-conserved positions due to minor structural differences are in blue boxes and are indicated by a period (.). The β strands are shown by green arrows above the ORF8 sequence while the spanning loops were represented by black lines (Flower et al, 2021). The FR3 region (shown in the yellow box) was defined according to the IMGT delimitations. (**B**) Comparison of the overlapping sequences of SARS-CoV-2 ORF8 and human IGLV genes highlighted in the dashed boxes using peptide properties: buried index, hydrophobicity, polarity, and turn and helix propensity. The legend for each property is shown as a gradient color scheme on the right. (**C**–**E**) Structures of ORF8 (**C**), IGLV5-39*01 (**D**), and superimposed images after pairwise structural alignment as viewed from the top (left) and side (right). PBD entries: 7JTL (SARS-CoV-2, blue) and 2CD0 (human IGLV5-39, green). Overlapping sequences between the two structures are highlighted in red. (**F**) Pairwise structural alignment of the human IgG-Fc region (cyan, PDB ID:1FC1) and ORF8 (in blue) showing close structural similarity at the CH2 domain (193 atoms aligned; RMSD: 5.9 Å). The CH2 and CH3 domains are highlighted, while residues in red sphere show the neonatal Fc receptor (FcRn) interaction site. (**G**) Superimposition of CH2 domain of the human IgG-Fc and ORF8 from different rotational perspectives. CH2 residues with the closest folding to ORF8 (blue) is shown in flesh while CH3 are in cyan. The glycosylation site at N297 at the CH2 interface is shown in cyan stick models.

 © The Author(s)
